# Supplementary material for: Structure, interdomain dynamics, and pH-dependent autoactivation of pro-rhodesain, the main lysosomal cysteine protease from African trypanosomes
Source: J Biol Chem. 2021 Mar 18;296:100565. doi: 10.1016/j.jbc.2021.100565 (PMC8080524; doi:10.1016/j.jbc.2021.100565)
Supplement: Supplemental Figures S1–S9 and Tbale S1–S7 [file mmc1.pdf]

Supporting Information for

Structure, interdomain dynamics and pH-dependent autoactivation of pro-rhodesain, the main lysosomal cysteine protease from African trypanosomes

Patrick Johé[1], Elmar Jaenicke[3], Hannes Neuweiler[2], Tanja Schirmeister[1], Christian Kersten[1,*], Ute A. Hellmich[4,5,6,7*]

[1]Institute of Pharmaceutical and Biomedical Sciences, Johannes Gutenberg-University, Mainz, Germany
[2]Department for Biotechnology and Biophysics, Julius-Maximilians-University, Würzburg, Germany
[3]Institute for Molecular Physiology, Johannes Gutenberg-University, Mainz, Germany
[4]Department of Chemistry, Biochemistry Division, Johannes Gutenberg-University, Mainz, Germany
[5]Centre for Biomolecular Magnetic Resonance (BMRZ), Goethe University, Frankfurt, Germany
[6]current address: Faculty of Chemistry and Earth Sciences, Institute of Organic and Macromolecular Chemistry, Friedrich-Schiller-University, Jena, Germany
[7]Cluster of Excellence 'Balance of the Microverse', Friedrich-Schiller-University, Jena, Germany

*To whom correspondence should be addressed:
Christian Kersten: Institute of Pharmaceutical and Biomedical Sciences, Johannes Gutenberg-University, Mainz, Germany: kerstec@uni-mainz.de; Tel.: (+49)-(0)6131-39-25714
or
Ute A. Hellmich: Faculty of Chemistry and Earth Sciences, Institute of Organic and Macromolecular Chemistry, Friedrich-Schiller-University, Jena, Germany: ute.hellmich@uni-jena.de; Tel.: (+49)-(0)3641-9- 48761

Extended Materials and Methods

Optimization of purification of pro- and mature rhodesain from E. coli

Initially, the pro-rhodesain construct used for heterologous expression in *E. coli* contained the N-terminal propeptide, the catalytic domain, a tobacco etch virus (TEV)-protease cleavage sequence and a C-terminal 6xHis tag (Fig. S1A). This construct differs from the wildtype protein as it is expressed in *T. brucei* by the lack of the first 20 amino acids constituting the signal peptide and a C-terminal domain of unknown function. While protein expression could be induced by IPTG, multiple bands were observed on SDS-PAGE in the expected size-range for (pro-)rhodesain (~37/~24 kDa) (Fig. S1B). Although western blot analysis with anti-His_6-HPR revealed that the 6xHis-tag remained attached to the protein, unexpectedly, no proper interaction with a Ni^{2+}-NTA matrix and protein purification could be achieved (Fig. S1C, D).

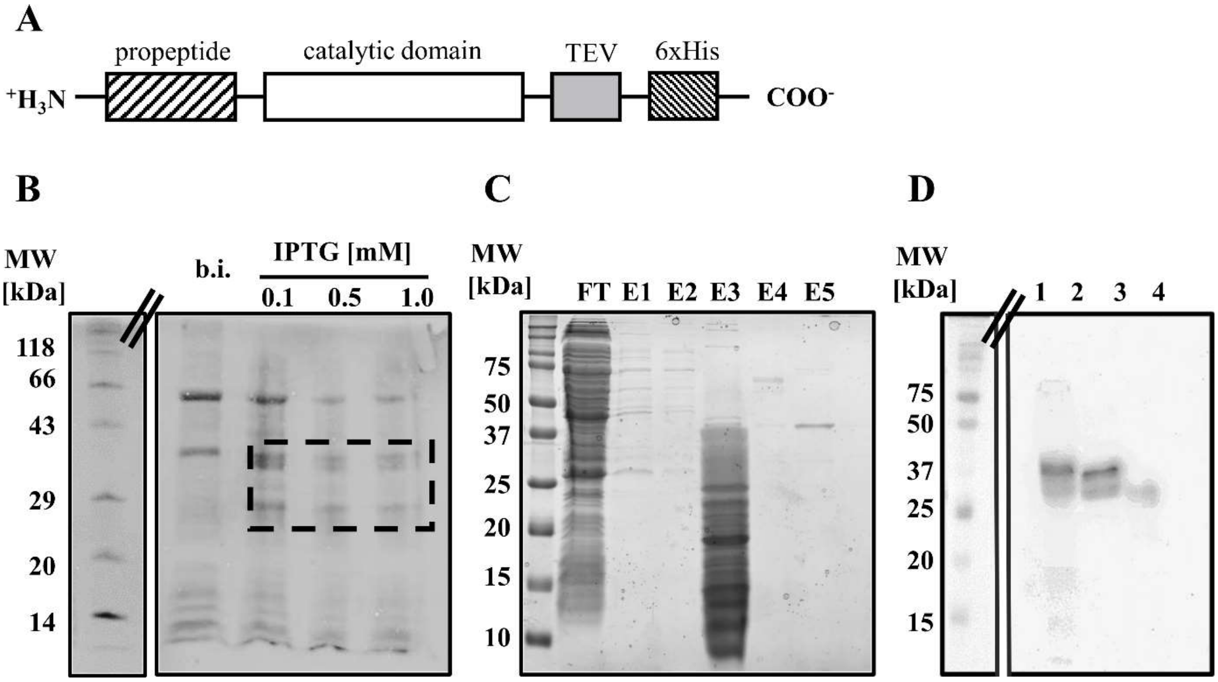

Figure S1: Expression and purification optimization of *T. b. rhodesiense* rhodesain from *E. coli*. A) Initial pro-rhodesain construct design. B) 15% SDS-PAGE of whole *E. coli* cell lysate before (b.i.) and after induction with various IPTG concentrations. Boxed area shows bands whose sizes correspond to the expected (pro)rhodesain protein. C) 15% SDS-PAGE of flow-through (FT) and different Ni^{2+}-NTA elution fractions during pro-rhodesain purification via IMAC. D) Western blot with anti-His_6-antibody of the soluble fraction of *E. coli* cells shows that full-length pro-rhodesain had been successfully expressed and that the C-terminal His-tag remained attached to the protein. (Cells were lysed in the presence of different additives (1: lysis buffer; 2: lysis buffer + 0.5% (v/v) TritonX; 3: lysis buffer + 1 M urea; 4: lysis buffer treatment of cells without IPTG induction).

To tackle this issue, we incorporated a green fluorescent protein (GFP) between the TEV cleavage sequence and the 6xHis-tag to increase the distance between the tag and the protein, to improve solubility and to be able to follow the purification progress more easily (Fig. S2A). The DNA and peptide sequences of the rhodesain-GFP construct are shown in Figure S3. Indeed, insertion of GFP led to a construct that was efficiently expressed and could be purified with Ni^{2+}-NTA beads (Fig. S2B). TEV protease efficiently cleaved the GFP-His6 tag, however, the separation of pro-rhodesain and the GFP by

an additional Ni^{2+}-NTA based IMAC purification step failed because both fragments were found in the flow through. A more complicated purification scheme thus had to be established (see below). However, for the inactive pro-rhodesain C150A constructs, this was not the case and these constructs could be purified efficiently by combining an IMAC purification step with a TEV digest, a second "reverse" IMAC and a SEC step (see main paper for details). This indicates that loss of the GFP-6xHis fragment's ability to interact with the Ni^{2+}-NTA matrix may be related to an inherent, weak rhodesain activity.

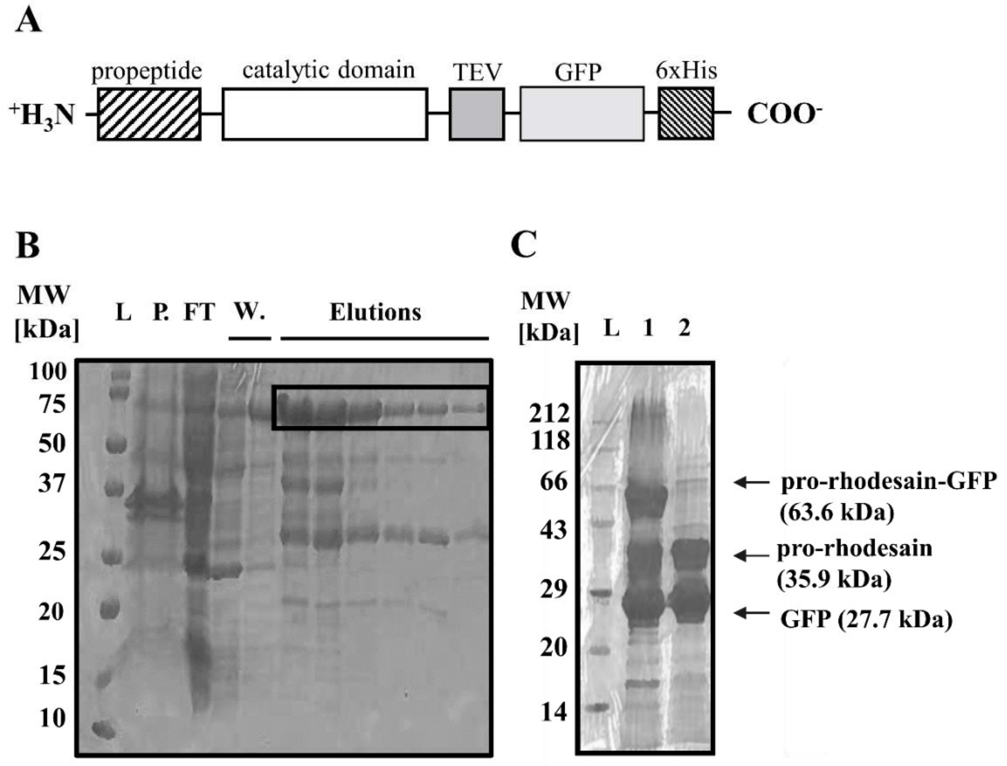

Figure S2: Insertion of GFP in the (pro-)rhodesain construct enables purification by immobilized metal affinity chromatography (IMAC). A) Construct design. B) 15% SDS-PAGE of different elution fraction from the purification of pro-rhodesain-GFP via IMAC. Protein marker (L.), insoluble fraction (P.), flow through (FT), wash fraction (at 20 mM imidazole, W.) and elution fractions (at 100 mM imidazole) are marked. The bands of the pro-rhodesain-GFP fusion protein are boxed. C) 15% SDS-PAGE of the Ni^{2+}-NTA elution fraction before (1) and after (2) cleavage by TEV protease.

Furthermore, because the GFP-His6-tag and the protease could not be separated efficiently by IEX or SEC after TEV cleavage, the mature wildtype protease had to be purified taking advantage of its auto-activation behavior. A drop in pH was used to activate the autocleavage of the enzyme, which simultaneously led to the digestion as well as the slow denaturation of the released GFP and thereby to the vanishing of the green color in the solution. Precipitated GFP was removed by centrifugation. Although the rhodesain activation step contributed to purification of the protease itself, it needed to be carefully controlled, due to the occurrence of autoproteolysis products, which occur after the enzyme reaches a short plateau of maximum catalytic activity (Fig. S4A, B). As described in the main paper, autoproteolysis can be prevented via the reversible inhibition of the enzyme with PMSF. Once the propeptide was removed, the released mature rhodesain could be purified from the remaining peptide

fragments by SEC. Alternatively final purification could also be achieved by anion exchange chromatography, due to the very low pI value of 3.94 for mature rhodesain.

```
ATGGCTTGTCTAGCATCAGTAGCTCTAGGGAGTTTACACGTTGAAGAATCACTAGAAATGCGTTTT
 M  A  C  L  A  S  V  A  L  G  S  L  H  V  E  E  S  L  E  M  R  F
GCGGCGTTCAAGAAGAAATACGGTAAGGTGTACAAAGACGCGAAGGAAGAGGCGTTCCGTTTTCGT
 A  A  F  K  K  K  Y  G  K  V  Y  K  D  A  K  E  E  A  F  R  F  R
GCGTTCGAGGAAAACATGGAGCAAGCGAAAATCCAAGCGGCGGCGAACCCGTATGCGACCTTCGGC
 A  F  E  E  N  M  E  Q  A  K  I  Q  A  A  A  N  P  Y  A  T  F  G
GTTACCCCGTTTAGCGATATGACCCGTGAGGAATTCCGTGCGCGTTACCGTAACGGTGCGAGCTAT
 V  T  P  F  S  D  M  T  R  E  E  F  R  A  R  Y  R  N  G  A  S  Y
TTTGCGGCGGCGCAAAAACGTCTGCGTAAGACCGTGAACGTTACCACCGGTCGTGCGCCGGCGGCG
 F  A  A  A  Q  K  R  L  R  K  T  V  N  V  T  T  G  R  A  P  A  A
GTGGACTGGCGTGAAAAGGGTGCGGTGACCCCGGTTAAGGATCAGGGCCAATGCGGTAGCTGCTGG
 V  D  W  R  E  K  G  A  V  T  P  V  K  D  Q  G  Q  C  G  S  C  W
GCGTTCAGCACCATCGGCAACATTGAGGGCCAGTGGCAAGTGGCGGGCAACCCGCTGGTTAGCCTG
 A  F  S  T  I  G  N  I  E  G  Q  W  Q  V  A  G  N  P  L  V  S  L
AGCGAACAGATGCTGGTGAGCTGCGACACCATCGATTTCGGTTGCGGTGGCGGTCTGATGGACAAC
 S  E  Q  M  L  V  S  C  D  T  I  D  F  G  C  G  G  G  L  M  D  N
GCGTTTAACTGGATTGTGAACAGCAACGGCGGTAACGTTTTTACCGAGGCGAGCTACCCGTATGTT
 A  F  N  W  I  V  N  S  N  G  G  N  V  F  T  E  A  S  Y  P  Y  V
AGCGGCAACGGCGAGCAGCCGCAATGCCAGATGAACGGCCACGAAATCGGTGCGGCGATTACCGAC
 S  G  N  G  E  Q  P  Q  C  Q  M  N  G  H  E  I  G  A  A  I  T  D
CACGTGGATCTGCCGCAAGACGAGGATGCGATTGCGGCGTACCTGGCGGAAAACGGTCCGCTGGCG
 H  V  D  L  P  Q  D  E  D  A  I  A  A  Y  L  A  E  N  G  P  L  A
ATTGCGGTTGATGCGACCAGCTTTATGGATTATAACGGCGGTATTCTGACCAGCTGCACCAGCGAA
 I  A  V  D  A  T  S  F  M  D  Y  N  G  G  I  L  T  S  C  T  S  E
CAGCTGGACCACGGCGTGCTGCTGGTTGGTTACAACGATGCGAGCAACCCGCCGTATTGGATCATT
 Q  L  D  H  G  V  L  L  V  G  Y  N  D  A  S  N  P  P  Y  W  I  I
AAAAACAGCTGGAGCAACATGTGGGGCGAGGATGGTTACATCCGTATTGAAAAGGGCACCAACCAA
 K  N  S  W  S  N  M  W  G  E  D  G  Y  I  R  I  E  K  G  T  N  Q
TGCCTGATGAACCAGGCGGTTAGCAGCGCGGTTGTTGGCGGCCCGGAGAATCTGTATTTTCAAGGT
 C  L  M  N  Q  A  V  S  S  A  V  V  G  G  P  E  N  L  Y  F  Q ‖G
AGCGTGAGCAAGGGCGAGGAGCTGTTCACCGGGGTGGTGCCCATCCTGGTCGAGCTGGACGGCGAC
 S  V  S  K  G  E  E  L  F  T  G  V  V  P  I  L  V  E  L  D  G  D
GTAAACGGCCACAAGTTCAGCGTGTCCGGCGAGGGCGAGGGCGATGCCACCTACGGCAAGCTGACC
 V  N  G  H  K  F  S  V  S  G  E  G  E  G  D  A  T  Y  G  K  L  T
CTGAAGTTCATCTGCACCACCGGCAAGCTGCCCGTGCCCTGGCCCACCCTCGTGACCACCCTGACC
 L  K  F  I  C  T  T  G  K  L  P  V  P  W  P  T  L  V  T  T  L  T
TACGGCGTGCAGTGCTTCAGCCGCTACCCCGACCACATGAAGCAGCACGACTTCTTCAAGTCCGCC
 Y  G  V  Q  C  F  S  R  Y  P  D  H  M  K  Q  H  D  F  F  K  S  A
ATGCCCGAAGGCTACGTCCAGGAGCGCACCATCTTCTTCAAGGACGACGGCAACTACAAGACCCGC
 M  P  E  G  Y  V  Q  E  R  T  I  F  F  K  D  D  G  N  Y  K  T  R
GCCGAGGTGAAGTTCGAGGGCGACACCCTGGTGAACCGCATCGAGCTGAAGGGCATCGACTTCAAG
 A  E  V  K  F  E  G  D  T  L  V  N  R  I  E  L  K  G  I  D  F  K
GAGGACGGCAACATCCTGGGGCACAAGCTGGAGTACAACTACAACAGCCACAACGTCTATATCATG
 E  D  G  N  I  L  G  H  K  L  E  Y  N  Y  N  S  H  N  V  Y  I  M
GCCGACAAGCAGAAGAACGGCATCAAGGTGAACTTCAAGATCCGCCACAACATCGAGGACGGCAGC
 A  D  K  Q  K  N  G  I  K  V  N  F  K  I  R  H  N  I  E  D  G  S
GTGCAGCTCGCCGACCACTACCAGCAGAACACCCCCATCGGCGACGGCCCCGTGCTGCTGCCCGAC
 V  Q  L  A  D  H  Y  Q  Q  N  T  P  I  G  D  G  P  V  L  L  P  D
AACCACTACCTGAGCACCCAGTCCGCCCTGAGCAAAGACCCCAACGAGAAGCGCGATCACATGGTC
 N  H  Y  L  S  T  Q  S  A  L  S  K  D  P  N  E  K  R  D  H  M  V
CTGCTGGAGTTCGTGACCGCCGCCGGGATCACTCTCGGCATGGACGAGCTGTACAAGCACCACCAT
 L  L  E  F  V  T  A  A  G  I  T  L  G  M  D  E  L  Y  K  H  H  H
CACCACCACTAA
 H  H  H ***
```

Figure S3: DNA and peptide sequences of the rhodesain-GFP construct. The stop codon (***), propeptide (<u>underlined</u>), C150A mutation (<u>underlined</u>), TEV cleavage site (‖) and GFP (<u>underlined</u>) are highlighted.

Detailed expression and purification protocol of pro-rhodesain: *E. coli* Rosetta2 DE3 pLysS cells were transformed with the plasmid encoding the GFP-tagged pro-rhodesain were grown at 37 °C in 100 mL LB medium in the presence of 100 µg/mL ampicillin and 50 µg/mL chloramphenicol overnight. 3 L main culture (LB medium, 100 µg/mL ampicillin, 50 µg/mL chloramphenicol) were inoculated with 2 Vol% of pre-culture and incubated at 37 °C. When the culture reached an OD_{600} of ~ 0.5 after 5 h, IPTG was added to a final concentration of 0.5 mM for induction. The cells were cultivated at 19.5 °C for 18 h, harvested by centrifugation at 15,000 *g* at 4 °C and stored at -80 °C.

The cell pellet was resuspended in 100 mL lysis buffer containing 50 mM Tris pH 8.5, 100 mM NaCl, 10 mM imidazole and 1 mM benzamidine. The cells were incubated for 15 min on ice in the presence of lysozyme and DNase I. After sonication on ice, the non-soluble cell fragments were sedimented by centrifugation at 25,000 *g* for 30 min at 4 °C and the green supernatant was loaded onto a Ni-NTA column with gravity flow. After a first washing step with 20 mM imidazole the green rhodesain-GFP construct was eluted at 100 mM imidazol concentration. 10% TEV-protease (n/n) was added to the combined fractions containing the desired protein to cleave the cysteine protease from its fusion tag. The solution was dialyzed against 50 mM Tris pH 8, 0.5 mM EDTA, 3 mM Glutathion red., 0.3 mM Glutathion ox., 10 mM NaCl over night at 4 °C. The TEV-protease was removed by anion exchange chromatography (HiTrap Q 5 mL, Buffer A: 50 mM Tris pH 8 Buffer B: 50 mM Tris pH 8, 500 mM NaCl). However, a separation of pro-rhodesain and GFP could not be achieved at this step. The maturation of the rhodesain zymogen was induced by a pH drop. DTT was added to a final concentration of 2 mM prior to adding 200 mM citrate buffer (pH 3) dropwise until a final pH value of 3.5 was reached. The enzyme was incubated at room temperature and its activity monitored by a fluorometric enzyme assay until a maximum of activity was observed. DTT was eliminated using KTT (5 mM final concentration) and the enzyme was inhibited by adding 2 mM PMSF to prevent autohydrolysis. The resulting pro-rhodesain was concentrated by spin filtration and further purified by SEC (HiLoad 16/600 Superdex 75 pg column (GE Healthcare), 0.5 mL/min, 20 mM sodium citrate, pH 5.0, 200 mM NaCl). The protein was dialyzed against H_2O at 4 °C for 4 h, lyophilized and stored at -80 °C upon further use.

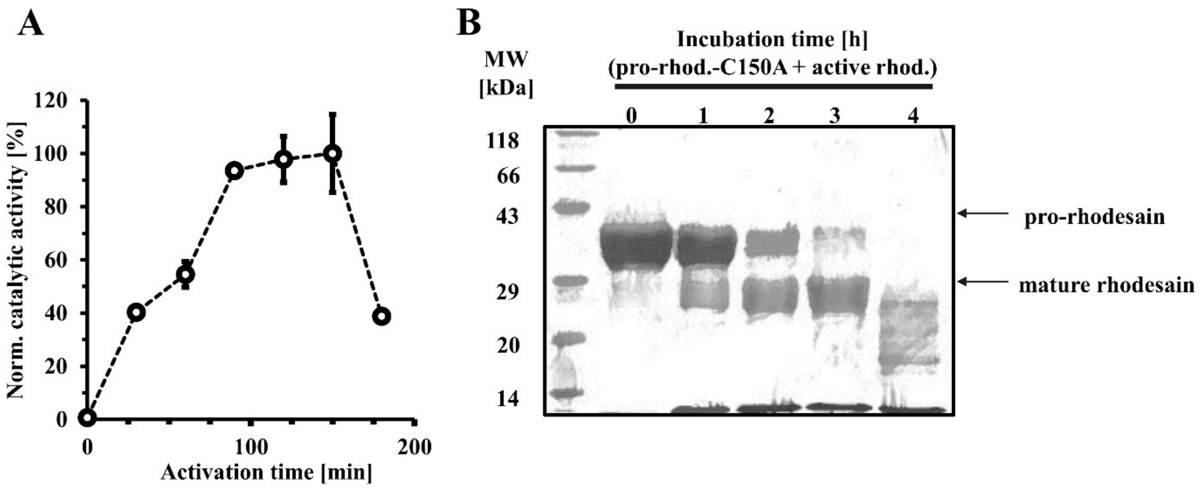

Figure S4: Autoactivation of rhodesain can lead to an unspecific autoproteolysis and an irreversible destruction of the enzyme. A) Autoactivation of pro-rhodesain was induced by a pH drop. Samples were taken at given time points and the catalytic activity determined via a fluorescence based enzyme assay with the peptidic substrate Z-Phe-Arg-AMC. The slope of the increasing fluorescence over time (which corresponds to the catalytic activity) was plotted against the pre-incubation time in the acidic milieu. B) SDS-PAGE of inactive pro-rhodesain C150A with active mature rhodesain shows complete digestion of enzymes at prolonged time points.

Site directed mutagenesis

Site directed mutagenesis was performed by Quik-change PCR with the KAPA-polymerase kit (KAPA HiFi HotStart PCR Kit, *Roche*, Mannheim, Germany). Primers were purchased from *Biomers* (Ulm, Deutschland) or IDT (*Integrated DNA Technologies*, Iowa, USA). The following primers were used to introduce point mutations into pro-rhodesain:

Q146W: 5′-CAGGGCTGGTGCGGTAGCTGCTGGGCGTTCAG-3′, 5′-CCGCACCAGCCCTGATCCTTAAC-CGGGGTCAC-3′; C150A: 5′-GCGGTAGCGCGTGGGCGTTCAGCACC-3′, 5'-CGCCCACGCGCTACCG-CATTGGCCCTG-3'; C22S: 5′-GGCTAGCCTAGCATCAGTAGCTCTAGGGAG-3′, 5′-CTGATGCTAGG-CTAGCCATATGTATATCTCCTTC-3′; A79C: 5′-GAATTCCGTTGCCGTTACCGTAACGGTGCG-3′, 5′-GTAACGGCAACGGAATTCCTCACGGGTCATATCGC-3′; V51C: 5′-CGGTAAGTGCTACAAAGA-CGCGAAGGAAGAGG-3′, 5′-CTTTGTAGCACTTACCGTATTTCTTCTTGAACGCCG-3′; D194N: 5′-CGGTCTGATGAACAACGCGTTTAACTGG-3′, 5′-GTTGTTCATCAGACCGCCACCGCAAC-3′; D242N: 5′-CCACGTGAACCTGCCGCAAGACGAGGATGC-3′, 5′-CGGCAGTTGCACGTGGTCGGTA-ATCGCCG-3′; Q146W (in C150A background): 5′-GATCAGGGCTGGTGCGGTAGCTGCTGGG-3′ and 5′-CGCACCAGCC-CTGATCCTTAACCGGGG-3′.

Fluorescence based cleavage assay

The catalytic activity of rhodesain was measured by detecting the hydrolysis of the fluorogenic substrate Z-Phe-Arg-AMC (Bachem) on a fluorimeter (F2000, fTecan). The enzyme (90 ng/mL) was incubated in 195 µL assay buffer (50 mM NaOAc pH 5.5, 200 mM NaCl, 5 mM EDTA, 5 mM DTT, 0.005% Brij35 (w/w)) at room temperature for 10 min before the reaction was started by adding 5 µL of substrate (varying concentrations in DMSO). The reaction was kept at 25 °C and the fluorescence of the released AMC was measured at a wavelength of 460 nm after excitation at a wavelength of 380 nm. Since the fluorescence intensity is proportional to the released AMC, the catalytic activity was derived from the slope of the line. Enzyme kinetic values K_M and v_{max} were calculated by GraFit® (5.0.13, Erithacus Software) and the fitting equation

$$v = \frac{v_{max}\,[S]}{K_M + [S]}$$

where v equals the catalytic activity at a given substrate concentration [S] and K_M is the Michaelis-Menten constant.

Comparison of the catalytic activities of mature rhodesain purified from *E. coli* or *P. pastoris* was probed using the fluorescence assay described above. The resulting K_M and v_{max} values for rhodesain expressed in *P. pastoris* are 5.78 ± 0.5 µM and 6.88 ± 0.7 s^{-1} and agree with those previously published[1,2]. Likewise, the values obtained for rhodesain purified from *E. coli* are 4.62 ± 0.42 µM and 5.47 ± 0.45 s^{-1} and thus show that the different expression host does not affect rhodesain functional integrity.

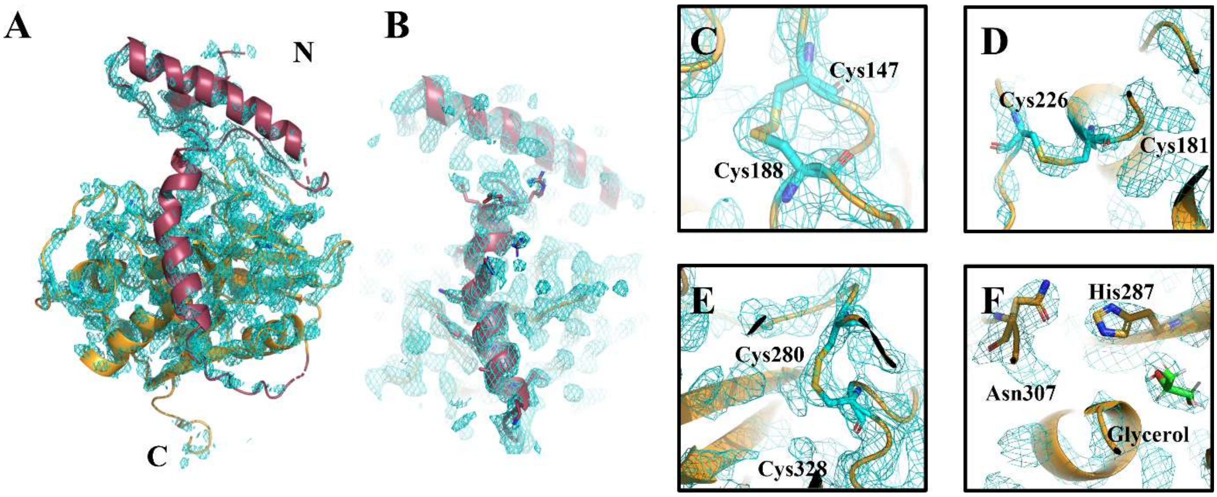

Figure S5: Electron density map of the pro-rhodesain crystal structure. The electron density map for the whole structure (A) and the pro-peptide (B) is colored in cyan at a sigma level of 2.0. The zoom-ins show the regions of the three disulfide bridges (C-E) and the active site (F).

Alignment of CathL zymogens from different species

```
Rhodesain       -----------ACLASVALGSLHVEESLEMRFAAFKKKYGKVYKDAKEEAFRFRAFEENM      69
Cruzain         ------------CLVPAATASLHAEETLTSQFAEFKQKHGRVYESAAEEAFRLSVFRENL      66
hsCathL         --------------------TLTFDHSLEAQWTKWKAMHNRLYGM-NEEGWRRAVWEKNM      56
Caricain        LFVHMSVSFGDFSIVGYSQDDLTSTERLIQLFNSWMLNHNKFYENVDEKLYRFEIFKDNL      76
Papain          --VYMGLSFGDFSIVGYSQNDLTSTERLIQLFESWMLKHNKIYKNIDEKIYRFEIFKDNL      76
                                     *   . *   :  :   :.:.*    *: :*   :..*:

                                                 blocking peptide
Rhodesain       EQAKIQAAAN----PYATFGVTPFSDMTREEFRARYRNGASYFA-AAQKRLRKTVNVTTG      124
Cruzain         FLARLHAAAN----PHATFGVTPFSDLTREEFRSRYHNGAAHFA-AAQERARVPVKVEVV      121
hsCathL         KMIELHNQEYREGKHSFTMAMNAFGDMTSEEFRQVMNGFQNRK----PRKGKVFQEPLFY      112
Caricain        NYIDETNKKN----NSYWLGLNEFADLSNDEFNEKYVGSLIDATIE-QSYDEEFINEDTV      131
Papain          KYIDETNKKN----NSYWLGLNVFADMSNDEFKEKYTGSIAGNYTTTELSYEEVLNDGDV      132
                                  :.:. *.*:: :**.    .             .   :

Rhodesain       RAPAAVDWREKGAVTPVKDQGQCGSCWAFSTIGNIEGQWQVAGNPLVSLSEQMLVSCDT-      183
Cruzain         GAPAAVDWRARGAVTAVKDQGQCGSCWAFSAIGNVECQWFLAGHPLTNLSEQMLVSCDK-      180
hsCathL         EAPRSVDWREKGYVTPVKNQGQCGSCWAFSATGALEGQMFRKTGRLISLSEQNLVDCSGP      172
Caricain        NLPENVDWRKKGAVTPVRHQGSCGSCWAFSAVATVEGINKIRTGKLVELSEQELVDCER-      190
Papain          NIPEYVDWRQKGAVTPVKNQGSCGSCWAFSAVVTIEGIIKIRTGNLNEYSEQELLDCDR-      191
                  *  **** :* ** *:.**.********:   :*         * . *** *:.*.

Rhodesain       -IDFGCGGGLMDNAFNWIVNSNGGNVFTEASYPYVSGNGEQPQCQMNGHEIGAAITDHVD      242
Cruzain         -TDSGCSGGLMNNAFEWIVQENNGAVYTEDSYPYASGEGISPPCTTSGHTVGATITGHVE      239
hsCathL         QGNEGCNGGLMDYAFQYVQ--DNGGLDSEESYPYEATE---ESCKYNPKYSVANDTGFVD      227
Caricain        -RSHGCKGGYPPYALEYVAK---NGIHLRSKYPYKAKQGTCRAKQVGGPIVKTSGVG-RV      245
Papain          -RSYGCNGGYPWSALQLVAQ---YGIHYRNTYPYEGVQRYCRSREKGPYAAKTDGVR-QV      246
                  . ** **    *:: :       :  . .*** . :        .     :  .

Rhodesain       LPQDEDAIAAYLAENGPLAIAVDA--TSFMDYNGGIL--TSCTSEQLDHGVLLVGYN---      295
Cruzain         LPQDEAQIAAWLAVNGPVAVAVDA--SSWMTYTGGVM--TSCVSEQLDHGVLLVGYN---      292
hsCathL         IPKQEKALMKAVATVGPISVAIDAGHESFLFYKEGIYFEPDCSSEDMDHGVLVVGYGFES      287
Caricain        QPNNEGNLLN-AIAKQPVSVVVESKGRPFQLYKGGIFEGP--CGTKVDHAVTAVGYG---      299
Papain          QPYNEGALLY-SIANQPVSVVLEAAGKDFQLYRGGIFVGP--CGNKVDHAVAAVGYG---      298
                 * :*  :        *:::.:::    :  *  *:       . .:**.*  ***.

Rhodesain       -DSSNPPYWIIKNSWSNMWGEDGYIRIEKGT-NQ---CLMNQAVSSAVVGGPTPPPPPP-      349
Cruzain         -DSAAVPYWIIKNSWTTQWGEEGYIRIAKGS-NQ---CLVKEEASSAVVGGPGPTPEPTT      347
hsCathL         TESDNNKYWLVKNSWGEEWGMGGYVKMAKDRRNH---CGIASAASYPTV-----------      333
Caricain        -KSGGKGYILIKNSWGTAWGEKGYIRIKRAPGNSPGVCGLYKSSYYPTKN----------      348
Papain          -----PNYILIKNSWGTGWGENGYIRIKRGTGNSYGVCGLYTSSFYPVKN----------      345
                       * ::****   **  **::: :   *    * :       .

Rhodesain       -------PPPSATFTQDFCEGKGCTKGCSHATFPTGECVQTTGVGSVIATCGASNLTQII      402
Cruzain         TTTTSAPGPSPSYFVQMSCTDAACIVGCENVTLPTGQCLLTTSGVSAIVTCGAETLTEEV      406
hsCathL         ------------------------------------------------------------      333
Caricain        ------------------------------------------------------------      348
Papain          ------------------------------------------------------------      345

Rhodesain       YPLSRSCSGLSVPITVPLDKCIPILIGSVEYHCSTNPPTKAARLVPHQ------------      450
Cruzain         FLTSTHCSGPSVRSSVPLNKCNRLLRGSVEFFCGSSSSGRLADVDRQRRHQPYHSRHRRL      467
hsCathL         ------------------------------------------------------------      333
Caricain        ------------------------------------------------------------      348
Papain          ------------------------------------------------------------      345
```

Figure S6: Alignment of cathepsin L-like proteases from different organisms. Amino acid sequences of *T. b. rhodesiense* pro-rhodesain (uniprot-ID.: Q95PM0), *T. cruzi* pro-cruzain (uniprot-ID.: P25779), *H. sapiens* pro-cathL (uniprot-ID.: P07711), *C. papaya* caricain and papain (uniprot-IDs: P10056, P00784) without the N-terminal signal peptides were aligned using the Clustal Ω multiple sequence alignment tool (version 1.2.4). X: conserved aromatic amino acids, cyan: ER(I/V)FNIN/ ER(A/V)FNAA motifs; yellow: GNFD motif, underlined: trypanosomal blocking peptide; green: β-sheet within the pro-peptide binding loop (PBL).

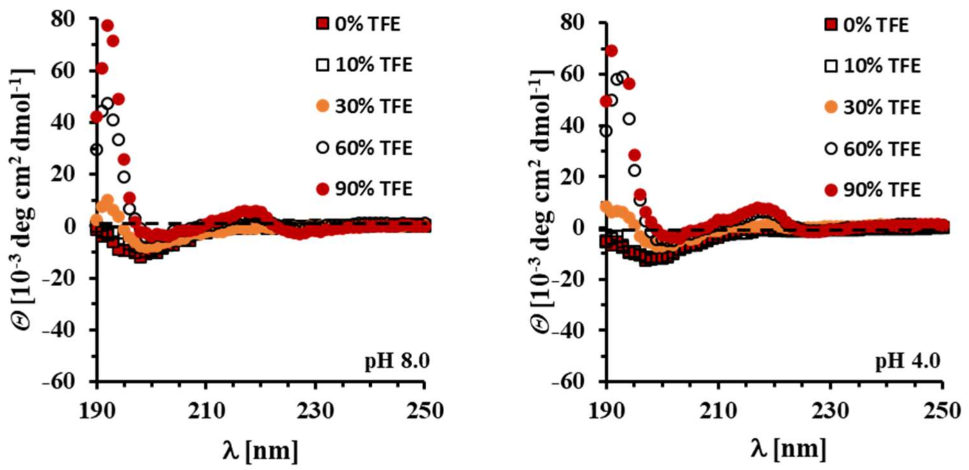

Figure S7: CD-spectra of the blocking peptide from human pro-CathL at various TFE concentrations and different pH values.

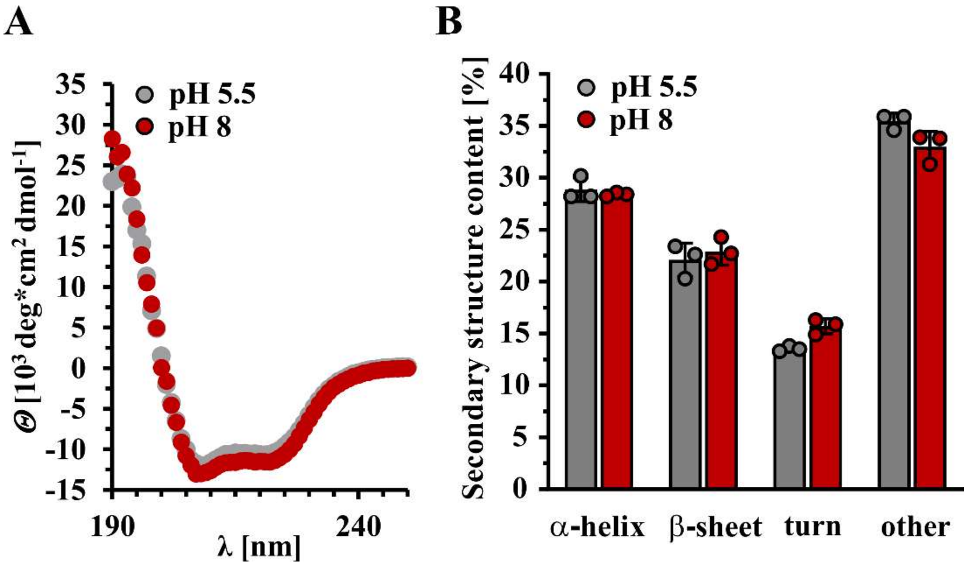

Figure S8: Comparison of secondary structure of pro-rhodesain at pH 8 and pH 5.5 (A) CD spectra of pro-rhodesain C150A at pH 8 and pH 5.5. (B) Calculated secondary structure content based on CD spectra using the BsStSel server.[3]

Molecular dynamics simulations

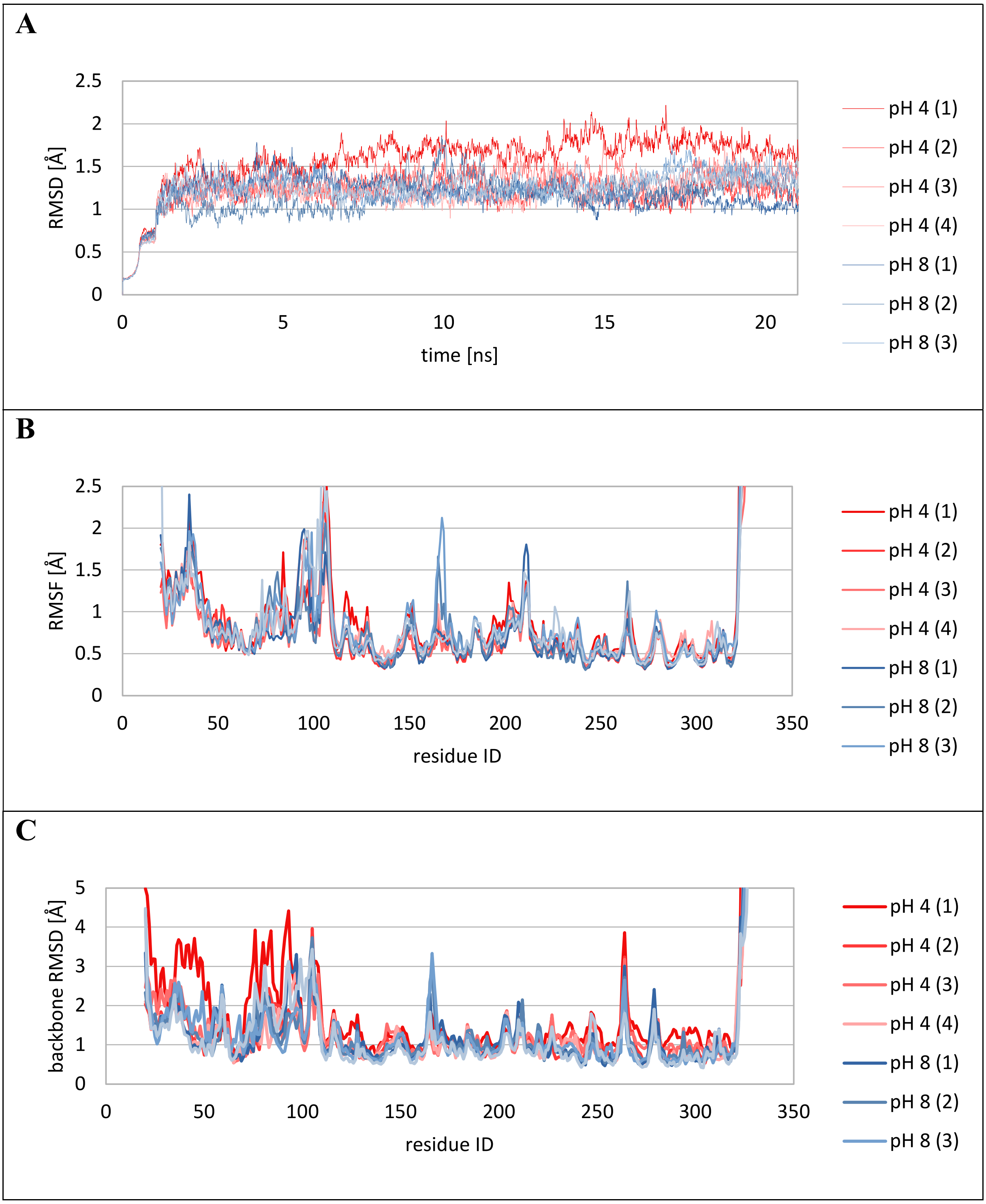

Figure S9: MD simulations on pro-rhodesain. A) 1-D RMSD traces of 8 MD simulations over 1 ns of equilibration and 20 ns production at pH 4 (red traces) and pH 8 (blue traces), respectively for backbone atoms excluding highly flexible C-terminal residues 338-348. B) C_α-RMSF per residue over 20 ns MD production run. C) RMSD per residue backbone compared to starting structure.

Table S1: Average intra- and interdomain electrostatic (E_{ele}) and van der Waals (E_{vdW}) energies from MD simulations within the pro-domain (pro), within the main domain (main), between pro-domain and main domain (pro-main), between pro-domain and solvent (pro-solv) and main domain and solvent (main-solv) calculated from MD simulations at pH 4 and 8, respectively. All values are in kcal/mol.

Simulation	E_{ele} (pro-main)	E_{vdW} (pro-main)	E_{ele} (pro)	E_{vdW} (pro)	E_{ele} (main)	E_{vdW} (main)	E_{ele} (pro-solv)	E_{vdW} (pro-solv)	E_{ele} (main-solv)	E_{vdW} (main-solv)
**pH 4 (1)**	-422.8	-226.5	-2697.9	-209.6	-5718.1	-900.0	-2703.8	-237.3	-3335.3	-340.8
**pH 4 (2)**	-359.4	-198.0	-2683.6	-213.5	-5701.7	-918.2	-2830.7	-255.6	-3482.1	-342.4
**pH 4 (3)**	-308.1	-190.4	-2654.3	-213.8	-5717.0	-919.4	-2915.3	-261.7	-3409.8	-324.8
**pH 4 (4)**	-264.5	-188.0	-2698.7	-220.1	-5654.9	-917.5	-2861.6	-256.3	-3715.7	-346.5
**pH 8 (1)**	-585.3	-198.3	-2731.4	-205.0	-5039.3	-894.7	-2933.7	-238.8	-4524.1	-294.2
**pH 8 (2)**	-706.4	-181.7	-2681.2	-214.1	-5042.2	-901.4	-2903.2	-241.8	-4605.8	-285.3
**pH 8 (3)**	-665.8	-191.4	-2811.4	-201.7	-5020.6	-886.7	-2676.1	-246.1	-4680.5	-302.4
**pH 8 (4)**	-678.2	-196.8	-2662.5	-206.6	-5092.5	-900.6	-2936.8	-234.5	-4563.2	-283.9

Table S2a: Relative occurrence of hydrogen bonds within the pro-domain during MD simulations. Four respective MD simulations at pH 4 (pH4_1 and pH4_2, see Table S2b for pH4_3 and pH4_4) were carried out. `Main' refers to interactions of the backbone. 'Side' indicates interactions of side chains. Occurrences below 5% were left out for clarity.

pH4_1							pH4_2						
						,							
donor			acceptor			occupancy	donor			acceptor			occupancy
TYR	52	Side	ASP	91	Side	70,00%	ARG	61	Side	GLU	96	Side	86,00%
ARG	61	Side	GLU	96	Side	66,45%	TYR	52	Side	ASP	91	Side	68,90%
GLN	75	Main	GLN	71	Main	50,65%	ARG	61	Side	ASP	91	Main	50,55%
ARG	61	Main	GLU	57	Main	49,55%	ARG	61	Main	GLU	57	Main	48,05%
ARG	61	Side	ASP	91	Main	45,45%	LYS	45	Main	PHE	41	Main	47,30%
LYS	45	Main	PHE	41	Main	45,35%	ARG	102	Main	ARG	98	Main	47,00%
ASN	79	Side	GLN	75	Main	45,05%	GLN	75	Main	GLN	71	Main	46,90%
GLH	66	Main	PHE	62	Main	38,75%	ARG	61	Side	GLU	57	Side	44,50%
GLH	58	Main	ASP	54	Main	36,25%	ASN	79	Side	GLN	75	Main	41,10%
PHE	108	Main	GLY	104	Main	36,15%	GLH	66	Main	PHE	62	Main	38,10%
ARG	61	Side	GLU	57	Side	33,35%	LYS	50	Side	ASP	91	Side	35,00%
LYS	47	Main	ALA	43	Main	33,20%	THR	93	Side	GLU	96	Side	34,65%
ALA	105	Main	TYR	101	Main	32,75%	THR	93	Main	GLU	96	Side	34,50%
ALA	64	Main	PHE	60	Main	31,20%	ALA	99	Main	GLH	95	Main	33,80%
THR	87	Side	SER	90	Side	31,05%	GLH	58	Main	ASP	54	Main	32,70%
ARG	100	Side	GLU	96	Side	29,90%	ALA	64	Main	PHE	60	Main	32,60%
ARG	98	Main	ARG	94	Main	28,95%	ALA	42	Main	GLH	38	Main	32,10%
ARG	100	Main	GLU	96	Main	28,80%	LYS	47	Main	ALA	43	Main	31,65%
ALA	42	Main	GLH	38	Main	28,55%	MET	92	Main	PHE	89	Main	31,20%
ASN	68	Side	SER	90	Main	28,30%	ARG	94	Side	GLU	67	Side	30,10%
PHE	97	Main	THR	93	Main	26,70%	PHE	97	Main	THR	93	Main	28,35%

MET	69	Main	PHE	65	Main	25,90%	GLN	71	Main	GLU	67	Main	27,70%
ARG	114	Main	ALA	110	Main	25,40%	ALA	72	Main	ASN	68	Main	26,95%
PHE	65	Main	ARG	61	Main	25,30%	LYS	56	Side	ASP	54	Side	26,40%
ASN	68	Side	MET	92	Main	24,75%	MET	69	Main	PHE	65	Main	24,60%
ARG	63	Main	ALA	59	Main	23,95%	LYS	56	Main	ASP	54	Side	23,60%
TYR	101	Main	PHE	97	Main	23,85%	THR	87	Main	SER	90	Side	23,25%
PHE	62	Main	GLH	58	Main	23,35%	PHE	65	Main	ARG	61	Main	22,30%
PHE	44	Main	ARG	40	Main	21,70%	ASN	68	Side	MET	92	Main	21,45%
LYS	46	Main	ALA	42	Main	21,45%	LYS	46	Main	ALA	42	Main	21,15%
THR	93	Side	GLU	67	Side	20,70%	PHE	44	Main	ARG	40	Main	20,20%
ALA	76	Main	ALA	72	Main	19,45%	ARG	63	Main	ALA	59	Main	19,95%
ALA	43	Main	MET	39	Main	16,75%	PHE	62	Main	GLH	58	Main	18,90%
LYS	50	Side	ASP	91	Side	16,55%	ILE	74	Main	GLU	70	Main	18,40%
LYS	73	Side	GLU	70	Side	16,55%	ALA	109	Main	ALA	105	Main	17,50%
GLN	71	Main	GLU	67	Main	16,25%	ASN	68	Side	SER	90	Main	17,30%
ALA	72	Main	ASN	68	Main	15,95%	ARG	114	Main	ALA	110	Main	16,90%
LYS	56	Main	ASP	54	Side	15,80%	ALA	76	Main	ALA	72	Main	16,55%
ALA	59	Main	ALA	55	Main	14,75%	ALA	43	Main	MET	39	Main	16,50%
LYS	50	Side	PRO	88	Main	14,45%	GLU	67	Main	ARG	63	Main	16,25%
ALA	109	Main	ALA	105	Main	13,45%	ALA	59	Main	ALA	55	Main	15,60%
GLH	58	Side	GLH	38	Side	12,85%	ALA	110	Main	SER	106	Main	15,25%
ARG	94	Side	GLU	67	Side	12,45%	ARG	98	Main	ARG	94	Main	14,40%
PHE	60	Main	LYS	56	Main	12,35%	LYS	73	Side	GLU	70	Side	13,55%
GLU	70	Main	GLH	66	Main	12,30%	PHE	60	Main	LYS	56	Main	13,30%
LYS	56	Side	ASP	54	Side	12,25%	ASN	68	Main	ALA	64	Main	12,20%
TYR	48	Main	PHE	44	Main	11,65%	TYR	48	Main	PHE	44	Main	11,95%
GLU	57	Main	ASP	54	Side	11,30%	ALA	77	Main	LYS	73	Main	11,20%
ASN	68	Main	ALA	64	Main	10,50%	ALA	111	Main	TYR	107	Main	11,20%
GLU	67	Main	ARG	63	Main	10,40%	ALA	78	Main	ILE	74	Main	11,10%
LYS	45	Side	GLH	58	Side	9,50%	ASN	68	Side	ALA	64	Main	10,70%
ILE	74	Main	GLU	70	Main	9,25%	ALA	82	Main	ASN	79	Main	10,15%
TYR	107	Main	ASN	103	Main	7,85%	LYS	50	Main	LYS	45	Main	9,95%
LYS	53	Side	ASP	54	Side	7,55%	GLN	112	Main	PHE	108	Main	9,70%
THR	93	Side	ASN	68	Side	7,50%	TYR	101	Main	ARG	98	Main	9,55%
LYS	50	Main	LYS	45	Main	6,45%	LYS	45	Side	GLH	58	Side	8,30%
SER	90	Main	THR	87	Main	6,45%	ALA	105	Main	ARG	102	Main	7,90%
LYS	113	Main	ALA	109	Main	5,80%	GLU	57	Main	ASP	54	Side	7,30%
ALA	77	Main	LYS	73	Main	5,40%	GLY	104	Main	TYR	101	Main	7,20%
							SER	106	Side	ASN	103	Main	7,00%
							ARG	100	Main	GLU	96	Main	6,55%
							GLU	96	Main	THR	93	Side	6,25%
							THR	93	Side	GLU	67	Side	6,10%

Table S2b: Relative occurrence of hydrogen bonds within the pro-domain. Four respective MD simulations at pH 4 (pH4_3 and pH4_4, see Table S2a for pH4_1 and pH4_2) were carried out. `Main' refers to interactions of the backbone. 'Side' indicates interactions of side chains. Occurrences below 5% were left out for clarity.

pH 4_3							pH 4_4						
donor			acceptor			occupancy	donor			acceptor			occupancy
ARG	61	Side	GLU	96	Side	88,31%	ARG	61	Side	GLU	96	Side	87,01%
TYR	52	Side	ASP	91	Side	76,31%	TYR	52	Side	ASP	91	Side	77,06%
ARG	61	Side	ASP	91	Main	52,82%	ARG	61	Side	GLU	57	Side	52,92%
LYS	45	Main	PHE	41	Main	50,52%	THR	93	Side	GLU	96	Side	49,78%
ARG	61	Side	GLU	57	Side	49,08%	LYS	45	Main	PHE	41	Main	49,53%
ARG	61	Main	GLU	57	Main	44,18%	ARG	61	Side	ASP	91	Main	48,38%
THR	93	Side	GLU	96	Side	42,18%	THR	93	Main	GLU	96	Side	45,63%
GLH	66	Main	PHE	62	Main	41,43%	ARG	61	Main	GLU	57	Main	43,48%
GLN	75	Main	GLN	71	Main	40,23%	GLH	66	Main	PHE	62	Main	41,23%
ASN	79	Side	GLN	75	Main	39,48%	ARG	102	Main	ARG	98	Main	40,73%
GLN	71	Main	GLU	67	Main	38,18%	ASN	68	Side	SER	90	Main	38,58%
MET	69	Main	PHE	65	Main	36,53%	ASN	68	Side	MET	92	Main	38,08%
THR	93	Main	GLU	96	Side	35,78%	GLN	75	Main	GLN	71	Main	36,18%
ASN	68	Side	SER	90	Main	35,13%	TYR	101	Main	PHE	97	Main	34,63%
ASN	68	Side	MET	92	Main	33,58%	ALA	42	Main	GLH	38	Main	33,68%
ALA	42	Main	GLH	38	Main	33,53%	LYS	47	Main	ALA	43	Main	33,13%
PHE	97	Main	THR	93	Main	32,68%	ASN	79	Side	GLN	75	Main	31,48%
LYS	50	Side	ASP	91	Side	30,68%	ALA	99	Main	GLH	95	Main	30,28%
LYS	47	Main	ALA	43	Main	30,03%	MET	69	Main	PHE	65	Main	29,29%
ALA	99	Main	GLH	95	Main	29,24%	ARG	98	Main	ARG	94	Main	27,44%
GLH	58	Main	ASP	54	Main	28,84%	GLH	58	Main	ASP	54	Main	26,34%
SER	106	Side	ASN	103	Main	27,64%	PHE	65	Main	ARG	61	Main	26,29%
ALA	64	Main	PHE	60	Main	25,94%	GLN	71	Main	GLU	67	Main	26,29%
THR	87	Main	SER	90	Side	23,89%	GLU	57	Main	ASP	54	Side	25,64%
PHE	65	Main	ARG	61	Main	23,84%	ARG	94	Side	GLU	67	Side	25,24%
ALA	72	Main	ASN	68	Main	23,49%	THR	87	Main	SER	90	Side	25,04%
LYS	46	Main	ALA	42	Main	23,24%	ALA	105	Main	TYR	101	Main	24,94%
MET	92	Main	PHE	89	Main	22,54%	ARG	63	Main	ALA	59	Main	24,74%
PHE	62	Main	GLH	58	Main	22,44%	LYS	50	Side	ASP	91	Side	24,39%
ARG	63	Main	ALA	59	Main	22,19%	ALA	64	Main	PHE	60	Main	23,89%
ASN	68	Main	ALA	64	Main	21,99%	MET	92	Main	PHE	89	Main	22,99%
ALA	105	Main	TYR	101	Main	21,84%	ALA	72	Main	ASN	68	Main	21,34%
GLN	71	Side	GLU	67	Side	21,24%	LYS	46	Main	ALA	42	Main	21,09%
ALA	76	Main	ALA	72	Main	20,84%	ALA	76	Main	ALA	72	Main	20,59%
ILE	74	Main	GLU	70	Main	20,14%	PHE	44	Main	ARG	40	Main	18,69%
PHE	44	Main	ARG	40	Main	20,09%	PHE	62	Main	GLH	58	Main	18,29%
SER	90	Side	THR	87	Main	19,24%	LYS	73	Side	GLU	70	Side	17,79%
ALA	43	Main	MET	39	Main	18,54%	PHE	60	Main	LYS	56	Main	17,39%
ARG	98	Main	ARG	94	Main	18,19%	ASN	68	Main	ALA	64	Main	17,14%
GLU	57	Main	ASP	54	Side	15,79%	ALA	43	Main	MET	39	Main	16,99%
ALA	59	Main	ALA	55	Main	15,54%	LYS	113	Main	ALA	109	Main	16,69%

ALA	109	Main	ALA	105	Main	15,19%	ALA	59	Main	ALA	55	Main	15,74%
ARG	94	Side	GLU	67	Side	14,74%	GLN	71	Side	GLU	67	Side	14,99%
PHE	60	Main	LYS	56	Main	13,44%	ILE	74	Main	GLU	70	Main	14,29%
ALA	111	Main	TYR	107	Main	13,44%	GLU	67	Main	ARG	63	Main	12,64%
LYS	73	Side	GLU	70	Side	13,34%	PHE	97	Main	THR	93	Main	12,09%
GLN	112	Main	PHE	108	Main	13,14%	ALA	77	Main	LYS	73	Main	11,49%
ALA	110	Main	SER	106	Main	13,14%	LYS	50	Main	LYS	45	Main	11,24%
LYS	56	Main	ASP	54	Side	12,74%	LYS	53	Side	ASP	54	Side	10,69%
ALA	77	Main	LYS	73	Main	12,44%	ALA	109	Main	ALA	105	Main	10,59%
LYS	50	Main	LYS	45	Main	11,74%	GLY	104	Main	ARG	100	Main	9,90%
GLU	67	Main	ARG	63	Main	11,59%	TYR	48	Main	PHE	44	Main	9,45%
PHE	108	Main	GLY	104	Main	10,34%	LYS	45	Side	GLH	58	Side	9,30%
ARG	114	Main	ALA	110	Main	9,95%	SER	106	Side	ASN	103	Main	9,25%
LYS	45	Side	GLH	58	Side	9,40%	ARG	114	Main	ALA	110	Main	8,95%
GLU	96	Main	THR	93	Side	9,00%	THR	123	Side	VAL	121	Main	7,65%
GLN	75	Side	GLN	71	Main	8,90%	LYS	56	Main	ASP	54	Side	7,50%
TYR	48	Main	PHE	44	Main	8,45%	GLN	112	Main	PHE	108	Main	6,80%
ARG	100	Main	GLU	96	Main	8,20%	PHE	108	Main	GLY	104	Main	6,45%
THR	123	Side	VAL	121	Main	5,45%	ALA	82	Main	ASN	79	Main	6,30%
LYS	50	Side	PRO	88	Main	5,00%	ALA	78	Main	ILE	74	Main	6,20%
							ALA	111	Main	PHE	108	Main	5,85%
							SER	106	Side	ARG	102	Main	5,05%

Table S3a: Relative occurrence of hydrogen bonds within the pro-domain. Four respective MD simulations at pH 8 (pH8_1 and pH8_2, see Table S3b for pH8_3 and pH8_4) were carried out. `Main' refers to interactions of the backbone. 'Side' indicates interactions of side chains. Occurrences below 5% were left out for clarity.

pH8_1							pH8_2						
Found			153			hbonds,	Found			165			hbonds,
donor			acceptor			occupancy	donor			acceptor			occupancy
ARG	102	Side	GLU	95	Side	71,90%	ARG	61	Side	GLU	96	Side	81,65%
TYR	52	Side	ASP	91	Side	67,00%	TYR	52	Side	ASP	91	Side	76,05%
ASN	68	Side	GLY	85	Main	50,45%	THR	93	Side	GLU	96	Side	52,45%
GLN	71	Main	GLU	67	Main	49,05%	ARG	61	Side	ASP	91	Main	49,90%
LYS	45	Side	GLU	58	Side	44,20%	ASN	68	Side	SER	90	Main	49,65%
LYS	45	Main	PHE	41	Main	41,55%	LYS	45	Main	PHE	41	Main	47,80%
ARG	61	Main	GLU	57	Main	38,45%	LYS	45	Side	GLU	58	Side	45,60%
ARG	40	Side	GLU	66	Side	37,80%	ARG	61	Main	GLU	57	Main	43,30%
ARG	61	Side	ASP	91	Main	36,50%	ARG	61	Side	GLU	57	Side	37,65%
ASN	79	Side	GLN	75	Main	34,70%	THR	93	Main	GLU	96	Side	35,80%
GLU	58	Main	ASP	54	Main	34,35%	ALA	42	Main	GLU	38	Main	35,40%
ARG	98	Main	ARG	94	Main	34,35%	MET	69	Main	PHE	65	Main	35,15%
ARG	61	Side	GLU	96	Side	33,85%	ASN	68	Side	MET	92	Main	35,05%
LYS	47	Main	ALA	43	Main	33,65%	GLN	75	Main	GLN	71	Main	34,60%
ALA	43	Main	MET	39	Main	33,10%	GLU	66	Main	PHE	62	Main	34,25%
LYS	73	Side	GLU	70	Side	31,40%	ARG	102	Side	GLU	95	Side	34,05%
SER	106	Side	ASN	103	Main	30,75%	ASN	79	Side	GLN	75	Main	33,45%
GLU	66	Main	PHE	62	Main	28,85%	THR	87	Main	SER	90	Side	32,60%

ARG	61	Side	GLU	57	Side	28,75%	LYS	47	Main	ALA	43	Main	29,20%
PHE	62	Main	GLU	58	Main	27,80%	PHE	62	Main	GLU	58	Main	29,10%
LYS	46	Main	ALA	42	Main	26,45%	PHE	65	Main	ARG	61	Main	28,40%
ALA	76	Main	ALA	72	Main	26,20%	LYS	50	Side	ASP	91	Side	28,25%
ARG	94	Side	GLU	67	Side	26,05%	ALA	105	Main	TYR	101	Main	28,05%
LYS	50	Side	ASP	91	Side	25,45%	ALA	43	Main	MET	39	Main	27,95%
ALA	42	Main	GLU	38	Main	25,40%	ARG	98	Main	ARG	94	Main	27,35%
THR	87	Main	SER	90	Side	23,85%	ALA	64	Main	PHE	60	Main	25,10%
ARG	94	Side	GLN	71	Side	23,55%	MET	92	Main	PHE	89	Main	23,40%
THR	93	Side	GLU	96	Side	23,30%	LYS	46	Main	ALA	42	Main	22,75%
PHE	65	Main	ARG	61	Main	22,90%	GLU	58	Main	ASP	54	Main	22,50%
ALA	99	Main	GLU	95	Main	22,40%	SER	106	Side	ASN	103	Main	22,25%
PHE	97	Main	THR	93	Main	18,35%	ALA	99	Main	GLU	95	Main	21,65%
ARG	63	Main	ALA	59	Main	17,60%	LYS	73	Side	GLU	70	Side	20,90%
ALA	72	Main	ASN	68	Main	16,90%	ALA	111	Main	TYR	107	Main	19,65%
MET	92	Main	PHE	89	Main	15,40%	PHE	108	Main	GLY	104	Main	19,00%
ARG	114	Main	ALA	110	Main	15,15%	PHE	97	Main	THR	93	Main	17,80%
ALA	77	Main	LYS	73	Main	15,10%	PHE	44	Main	ARG	40	Main	17,05%
THR	93	Main	GLU	96	Side	14,70%	ALA	72	Main	ASN	68	Main	16,85%
SER	90	Side	ASN	68	Side	14,30%	ARG	63	Main	ALA	59	Main	16,05%
PHE	108	Main	GLY	104	Main	14,25%	ALA	76	Main	ALA	72	Main	15,80%
LYS	50	Side	PRO	88	Main	13,25%	ALA	59	Main	ALA	55	Main	14,05%
GLN	75	Side	GLN	71	Main	12,60%	GLN	71	Main	GLU	67	Main	12,85%
ALA	111	Main	TYR	107	Main	12,30%	ARG	116	Side	ALA	111	Main	12,60%
ASN	68	Main	ALA	64	Main	11,35%	ALA	77	Main	LYS	73	Main	12,25%
LYS	56	Main	ASP	54	Side	11,25%	ARG	102	Main	ARG	98	Main	12,20%
LYS	50	Main	LYS	45	Main	11,15%	ARG	94	Side	GLU	67	Side	11,90%
ALA	64	Main	PHE	60	Main	10,50%	GLU	57	Main	ASP	54	Side	11,70%
THR	123	Side	VAL	121	Main	10,40%	LYS	50	Main	LYS	45	Main	11,60%
MET	69	Main	PHE	65	Main	10,35%	ASN	68	Main	ALA	64	Main	10,45%
ALA	59	Main	ALA	55	Main	9,95%	TYR	48	Main	PHE	44	Main	9,50%
TYR	48	Main	PHE	44	Main	9,30%	ARG	100	Side	ARG	100	Main	9,50%
GLU	57	Main	ASP	54	Side	9,20%	SER	106	Side	ARG	102	Main	9,20%
ARG	63	Side	GLU	67	Side	8,50%	ALA	78	Main	ILE	74	Main	8,90%
ARG	40	Main	GLU	38	Side	8,40%	ALA	109	Main	ALA	105	Main	8,35%
PHE	44	Main	ARG	40	Main	8,30%	LYS	53	Main	GLU	57	Side	8,30%
ARG	61	Side	ASP	91	Side	8,20%	PHE	60	Main	LYS	56	Main	7,60%
ALA	82	Main	ASN	79	Main	7,75%	ALA	82	Main	ASN	79	Main	6,75%
LYS	113	Main	ALA	109	Main	7,65%	ILE	74	Main	GLU	70	Main	6,45%
LYS	56	Side	ASP	54	Side	7,00%	ARG	63	Side	GLU	67	Side	6,40%
ALA	105	Main	TYR	101	Main	6,75%	ARG	40	Main	GLU	38	Side	6,35%
ALA	109	Main	ALA	105	Main	6,70%	TYR	107	Main	ASN	103	Main	5,30%
GLN	112	Side	PHE	108	Main	6,00%	GLN	112	Side	ALA	109	Main	5,10%
GLN	75	Main	GLN	71	Main	5,60%	GLU	67	Main	ARG	63	Main	5,05%
ALA	78	Main	ILE	74	Main	5,55%							
GLU	67	Main	ARG	63	Main	5,00%							

Table S3b: Relative occurrence of hydrogen bonds within the pro-domain. Four respective MD simulations at pH 8 (pH8_3 and pH8_4, see Table S3a for pH8_1 and pH8_2) were carried out. `Main' refers to interactions of the backbone. 'Side' indicates interactions of side chains. Occurrences below 5% were left out for clarity.

pH_8_3							pH8_4						
Found			166			hbonds,	Found			162			hbonds,
donor			acceptor			occupancy	donor			acceptor			occupancy
TYR	52	Side	ASP	91	Side	74,56%	ARG	61	Side	GLU	96	Side	85,41%
ARG	61	Side	GLU	96	Side	72,86%	TYR	52	Side	ASP	91	Side	78,61%
SER	90	Side	ASN	68	Side	66,12%	SER	90	Side	ASN	68	Side	58,27%
THR	93	Side	GLU	96	Side	62,82%	LYS	45	Main	PHE	41	Main	46,18%
THR	93	Main	GLU	96	Side	53,17%	ARG	61	Side	ASP	91	Main	45,53%
ARG	94	Side	GLU	67	Side	44,48%	GLN	75	Main	GLN	71	Main	45,13%
ARG	61	Main	GLU	57	Main	43,53%	LYS	45	Side	GLU	58	Side	43,53%
LYS	45	Side	GLU	58	Side	42,33%	ARG	40	Side	GLU	66	Side	41,63%
ALA	76	Main	ALA	72	Main	41,18%	ARG	61	Main	GLU	57	Main	40,43%
ARG	98	Side	GLU	95	Side	38,08%	ARG	61	Side	GLU	57	Side	39,63%
LYS	45	Main	PHE	41	Main	35,28%	ARG	98	Main	ARG	94	Main	36,33%
LYS	47	Main	ALA	43	Main	34,58%	ASN	79	Side	GLN	75	Main	34,23%
ALA	42	Main	GLU	38	Main	32,03%	GLU	66	Main	PHE	62	Main	32,98%
ARG	63	Side	GLU	67	Side	31,73%	GLN	71	Main	GLU	67	Main	32,83%
LYS	46	Main	ALA	42	Main	31,08%	ALA	72	Main	ASN	68	Main	32,03%
ARG	40	Side	GLU	66	Side	30,73%	GLU	58	Main	ASP	54	Main	31,93%
GLU	58	Main	ASP	54	Main	30,58%	THR	93	Side	GLU	96	Side	31,78%
ARG	61	Side	ASP	91	Main	29,49%	LYS	47	Main	ALA	43	Main	30,58%
ARG	63	Side	ARG	63	Main	29,14%	THR	93	Main	GLU	96	Side	29,69%
ARG	94	Main	GLU	67	Side	28,69%	ASN	68	Side	GLY	85	Main	28,19%
PHE	65	Main	ARG	61	Main	28,54%	PHE	62	Main	GLU	58	Main	28,04%
LYS	50	Side	ASP	91	Side	27,24%	PHE	65	Main	ARG	61	Main	27,59%
ARG	61	Side	GLU	57	Side	26,94%	ARG	98	Side	GLU	95	Side	26,79%
ALA	43	Main	MET	39	Main	25,74%	LYS	46	Main	ALA	42	Main	25,84%
ASN	79	Side	GLN	75	Main	25,04%	LYS	50	Side	ASP	91	Side	24,94%
PHE	97	Main	THR	93	Main	23,39%	SER	90	Main	THR	87	Main	23,84%
ARG	98	Main	ARG	94	Main	21,44%	MET	69	Main	PHE	65	Main	22,54%
ALA	99	Main	GLU	95	Main	20,99%	LYS	73	Side	GLU	70	Side	20,49%
ALA	105	Main	TYR	101	Main	19,94%	TYR	48	Main	PHE	44	Main	19,59%
LYS	56	Main	ASP	54	Side	19,44%	PHE	97	Main	THR	93	Main	19,14%
THR	87	Main	SER	90	Side	17,54%	TYR	101	Main	PHE	97	Main	18,54%
MET	92	Main	PHE	89	Main	17,34%	ALA	43	Main	MET	39	Main	17,19%
ALA	111	Main	TYR	107	Main	17,24%	ALA	76	Main	ALA	72	Main	16,89%
LYS	56	Side	ASP	54	Side	16,99%	ARG	63	Main	ALA	59	Main	14,84%
LYS	73	Side	GLU	70	Side	16,99%	GLY	104	Main	TYR	101	Main	14,84%
ARG	102	Side	GLU	95	Side	16,29%	MET	92	Main	PHE	89	Main	14,64%
GLU	67	Main	ALA	64	Main	15,64%	ALA	59	Main	ALA	55	Main	14,44%
SER	106	Side	ASN	103	Main	14,69%	ARG	114	Main	ALA	110	Main	13,89%
PHE	62	Main	GLU	58	Main	13,84%	ALA	64	Main	PHE	60	Main	12,94%
ALA	110	Main	SER	106	Main	13,54%	LYS	50	Main	LYS	45	Main	12,84%
GLN	75	Main	GLN	71	Main	12,69%	ARG	102	Main	ARG	98	Main	11,99%

LYS	50	Main	LYS	45	Main	12,34%	THR	87	Main	SER	90	Side	11,29%
SER	90	Main	THR	87	Main	12,19%	ALA	77	Main	LYS	73	Main	11,04%
PHE	108	Main	GLY	104	Main	11,14%	LYS	56	Main	ASP	54	Side	11,04%
ARG	102	Main	ARG	98	Main	10,94%	ALA	78	Main	ILE	74	Main	10,99%
ARG	102	Side	ARG	98	Main	10,69%	LYS	56	Side	ASP	54	Side	10,39%
ALA	109	Main	ALA	105	Main	10,19%	ALA	105	Main	ARG	102	Main	9,35%
ALA	59	Main	ALA	55	Main	10,04%	PHE	44	Main	ARG	40	Main	9,05%
GLN	71	Main	GLU	67	Main	9,45%	ALA	99	Main	GLU	95	Main	8,95%
GLN	112	Main	PHE	108	Main	9,25%	ALA	42	Main	GLU	38	Main	8,90%
PHE	44	Main	ARG	40	Main	9,20%	LYS	113	Main	ALA	109	Main	8,65%
ALA	72	Main	ASN	68	Main	9,15%	ALA	109	Main	ALA	105	Main	8,20%
GLU	70	Main	GLU	66	Main	9,15%	ALA	82	Main	ASN	79	Main	6,95%
GLN	112	Side	PHE	108	Main	9,15%	ARG	94	Side	GLU	67	Side	6,50%
THR	123	Side	THR	122	Main	8,65%	PHE	108	Main	GLY	104	Main	6,35%
LYS	45	Side	GLU	38	Side	8,10%	SER	106	Side	ASN	103	Main	6,25%
LYS	73	Side	GLU	70	Main	7,40%	ARG	116	Side	ALA	111	Main	5,75%
ALA	64	Main	PHE	60	Main	6,90%	GLU	57	Main	ASP	54	Side	5,70%
GLU	66	Main	PHE	62	Main	6,65%	GLN	112	Main	ALA	109	Main	5,65%
ALA	78	Main	ILE	74	Main	6,50%	ASN	79	Main	GLN	75	Main	5,50%
ARG	94	Side	GLU	70	Side	6,40%	PHE	60	Main	LYS	56	Main	5,40%
ARG	114	Main	ALA	111	Main	6,15%							
TYR	48	Main	PHE	44	Main	5,70%							
ALA	77	Main	LYS	73	Main	5,55%							
GLY	104	Main	TYR	101	Main	5,50%							
ARG	40	Main	GLU	38	Side	5,25%							
ARG	114	Main	ALA	110	Main	5,20%							
ASN	79	Main	GLN	75	Main	5,20%							
TYR	101	Main	ARG	98	Main	5,00%							

Table S4a: Relative occurrence of hydrogen bonds within the catalytic domain. Four respective MD simulations at pH 4 (pH4_1 and pH4_2, see Table S4b for pH4_3 and pH4_4) were carried out. `Main' refers to interactions of the backbone. 'Side' indicates interactions of side chains. Occurrences below 5% were left out for clarity.

**pH4_1**							**pH4_2**						
**donor**			acceptor			occupancy	donor			acceptor			occupancy
**ASH**	185	Side	ASP	182	Side	86,05%	ASH	185	Side	ASP	182	Side	78,20%
**THR**	267	Side	ASP	265	Side	77,00%	THR	267	Side	ASP	265	Side	78,00%
**GLH**	175	Side	GLU	211	Side	76,25%	ASH	194	Side	ASP	242	Side	74,20%
**THR**	155	Side	TRP	151	Main	75,65%	THR	155	Side	TRP	151	Main	72,20%
**TYR**	272	Side	GLY	275	Main	71,00%	TYR	272	Side	GLY	275	Main	69,40%
**TRP**	303	Main	ILE	321	Main	69,20%	TRP	303	Main	ILE	321	Main	68,70%
**SER**	174	Side	TYR	214	Main	67,40%	TYR	294	Side	PRO	127	Main	66,35%
**ASH**	194	Side	ASP	242	Side	64,60%	ARG	320	Side	GLU	315	Side	65,25%
**LYS**	306	Main	LEU	290	Main	61,10%	GLH	175	Side	GLU	211	Side	62,45%
**THR**	139	Side	GLU	160	Side	61,00%	GLH	283	Main	ASP	265	Side	60,75%
**VAL**	241	Main	SER	336	Main	60,00%	VAL	241	Main	SER	336	Main	57,20%

**GLH**	283	Main	ASP	265	Side	58,75%	PHE	197	Main	MET	193	Main	57,05%
**SER**	218	Side	GLU	222	Main	58,15%	LYS	306	Main	LEU	290	Main	56,75%
**TYR**	216	Side	GLN	144	Main	56,95%	ILE	156	Main	ALA	152	Main	54,90%
**PHE**	197	Main	MET	193	Main	54,80%	SER	174	Side	TYR	214	Main	54,90%
**ARG**	320	Side	GLU	315	Side	54,60%	SER	174	Main	PHE	209	Main	53,95%
**GLN**	162	Main	ASN	158	Main	53,70%	TRP	132	Main	VAL	292	Main	53,90%
**ILE**	156	Main	ALA	152	Main	52,45%	ASH	296	Main	ALA	128	Main	53,60%
**SER**	154	Side	VAL	289	Main	52,20%	SER	218	Side	GLU	222	Main	53,20%
**ARG**	133	Side	ASP	131	Side	52,10%	GLN	144	Side	CYX	147	Main	51,70%
**SER**	154	Main	CYM	150	Main	51,60%	VAL	217	Main	GLN	176	Side	51,65%
**LEU**	173	Main	GLU	160	Side	51,30%	CYX	280	Main	GLN	327	Side	51,30%
**SER**	174	Main	PHE	209	Main	50,40%	HIP	287	Side	ASN	307	Side	50,40%
**GLU**	160	Main	ILE	156	Main	50,15%	TYR	216	Side	GLN	144	Main	50,15%
**GLN**	144	Side	CYX	147	Main	49,90%	GLN	162	Main	ASN	158	Main	49,90%
**HIP**	287	Side	ASN	307	Side	48,95%	GLN	284	Main	ASP	265	Side	48,60%
**ALA**	266	Main	ASH	286	Side	48,60%	LYS	323	Main	PRO	301	Main	46,85%
**VAL**	217	Main	GLN	176	Side	45,35%	GLH	322	Main	LEU	277	Main	45,30%
**SER**	308	Side	GLN	144	Side	44,85%	LEU	173	Main	GLU	160	Side	44,65%
**SER**	298	Side	PRO	300	Main	44,70%	THR	238	Main	VAL	338	Main	43,95%
**SER**	180	Main	GLN	176	Main	44,25%	GLU	211	Main	SER	172	Main	43,95%
**GLH**	322	Main	LEU	277	Main	44,20%	ASN	168	Side	TRP	163	Main	43,75%
**ASH**	296	Main	ALA	128	Main	43,75%	GLN	162	Side	ASN	257	Main	43,05%
**TRP**	132	Main	VAL	292	Main	43,15%	SER	213	Side	THR	210	Side	43,05%
**VAL**	264	Main	HIP	287	Main	42,55%	SER	298	Side	PRO	300	Main	42,80%
**GLN**	176	Side	SER	218	Side	42,20%	SER	180	Main	GLN	176	Main	41,15%
**LEU**	291	Main	LEU	260	Main	41,85%	GLU	160	Main	ILE	156	Main	40,60%
**VAL**	289	Main	ILE	262	Main	41,40%	SER	154	Main	CYM	150	Main	40,10%
**LYS**	323	Main	PRO	301	Main	41,35%	GLY	234	Main	VAL	208	Main	39,95%
**SER**	213	Side	THR	210	Side	41,20%	ASN	202	Main	ASN	198	Main	39,90%
**SER**	282	Side	CYX	280	Main	40,35%	GLY	275	Main	GLU	315	Side	39,75%
**ALA**	337	Main	ASN	158	Side	40,15%	ASN	158	Side	SER	336	Side	39,60%
**ILE**	237	Main	GLY	206	Main	39,80%	LEU	291	Main	LEU	260	Main	39,45%
**CYX**	280	Main	GLN	327	Side	39,75%	GLN	176	Side	SER	218	Side	39,35%
**ASN**	158	Side	SER	336	Side	39,45%	ILE	237	Main	GLY	206	Main	39,05%
**GLY**	275	Main	GLU	315	Side	37,95%	SER	282	Side	CYX	280	Main	38,30%
**ASN**	168	Side	TRP	163	Main	37,90%	VAL	165	Main	GLY	161	Main	38,00%
**GLY**	324	Main	GLU	247	Side	37,90%	LEU	254	Main	ILE	250	Main	37,85%
**LEU**	254	Main	ILE	250	Main	37,60%	ILE	250	Main	ASP	246	Main	37,20%
**GLY**	234	Main	VAL	208	Main	37,40%	ALA	337	Main	ASN	158	Side	36,75%
**TRP**	303	Side	GLU	247	Side	36,60%	THR	139	Side	GLU	160	Side	36,45%
**VAL**	165	Main	GLY	161	Main	36,45%	VAL	264	Main	HIP	287	Main	35,20%
**ILE**	304	Main	GLY	293	Main	36,25%	VAL	289	Main	ILE	262	Main	35,05%
**GLU**	211	Main	SER	172	Main	36,20%	TRP	303	Side	GLU	247	Side	34,55%
**ASN**	202	Main	ASN	198	Main	36,15%	VAL	338	Main	ASP	239	Main	34,00%
**VAL**	338	Main	ASP	239	Main	36,15%	ASN	331	Main	ASN	326	Side	33,45%
**GLN**	162	Side	ASN	257	Main	36,05%	HIP	287	Main	VAL	264	Main	33,15%
**ILE**	250	Main	ASP	246	Main	34,50%	GLN	176	Side	SER	180	Side	32,20%

**GLN**	284	Main	ASP	265	Side	33,55%	THR	267	Main	ASP	265	Side	31,90%
**THR**	267	Main	ASP	265	Side	33,00%	GLY	324	Main	GLU	247	Side	31,70%
**ASN**	331	Main	ASN	326	Side	32,45%	ASH	286	Main	VAL	264	Main	31,65%
**THR**	238	Main	VAL	338	Main	32,30%	HIP	287	Side	CYM	150	Side	30,65%
**ILE**	305	Main	ILE	319	Main	32,20%	ASN	204	Side	ASN	207	Main	30,60%
**GLN**	176	Side	SER	180	Side	31,85%	GLN	144	Main	SER	308	Main	30,15%
**ASN**	273	Side	TYR	272	Main	31,80%	ILE	305	Main	ILE	319	Main	29,90%
**ASH**	286	Main	VAL	264	Main	31,25%	ILE	304	Main	GLY	293	Main	29,60%
**THR**	238	Side	GLY	341	Main	30,40%	TRP	163	Main	ILE	159	Main	27,65%
**ASN**	257	Main	TYR	253	Main	30,00%	SER	335	Main	ALA	261	Main	27,25%
**LYS**	142	Side	GLU	160	Side	29,85%	ILE	184	Main	ASP	182	Side	26,55%
**VAL**	292	Main	ILE	304	Main	29,25%	VAL	208	Main	ALA	235	Main	26,35%
**SER**	335	Main	ALA	261	Main	28,90%	ASN	326	Main	GLU	247	Side	26,25%
**LEU**	277	Main	ARG	320	Main	28,85%	LEU	260	Main	LEU	291	Main	26,20%
**SER**	336	Main	VAL	241	Main	28,70%	VAL	292	Main	ILE	304	Main	25,30%
**ASN**	204	Side	ASN	207	Main	27,95%	ASN	273	Side	TYR	272	Main	24,55%
**TRP**	313	Side	PHE	269	Main	26,90%	GLN	327	Side	THR	325	Side	24,10%
**SER**	180	Side	PRO	224	Main	26,75%	SER	336	Main	VAL	241	Main	23,55%
**GLN**	327	Side	THR	325	Side	26,50%	ASN	295	Main	TYR	302	Main	23,55%
**TRP**	163	Main	ILE	159	Main	26,30%	ASN	257	Main	TYR	253	Main	23,50%
**THR**	210	Main	GLH	232	Main	25,70%	CYX	188	Main	GLN	223	Side	22,05%
**LEU**	243	Main	VAL	334	Main	24,75%	GLY	340	Main	ALA	236	Main	21,80%
**VAL**	208	Main	ALA	235	Main	24,70%	ALA	137	Main	TRP	132	Main	20,95%
**ASN**	326	Main	GLU	247	Side	24,45%	TRP	313	Side	PHE	269	Main	20,90%
**ASN**	307	Main	GLY	317	Main	24,45%	THR	155	Main	TRP	151	Main	20,75%
**ILE**	184	Main	ASP	182	Side	24,30%	LEU	243	Main	VAL	334	Main	20,65%
**LEU**	260	Main	LEU	291	Main	23,05%	SER	180	Side	PRO	224	Main	20,50%
**ASN**	295	Main	TYR	302	Main	22,85%	VAL	130	Main	TYR	294	Main	20,25%
**HIP**	287	Side	CYM	150	Side	21,75%	LEU	290	Main	LYS	306	Main	20,15%
**TYR**	294	Main	VAL	130	Main	21,15%	SER	154	Side	ILE	262	Main	20,15%
**SER**	335	Side	ASP	242	Side	20,95%	LEU	277	Main	ARG	320	Main	20,05%
**PHE**	347	Main	GLH	343	Main	20,85%	ALA	252	Main	ASP	248	Main	19,65%
**SER**	149	Side	TYR	216	Side	20,75%	TRP	199	Main	ASN	195	Main	18,80%
**GLN**	144	Main	SER	308	Main	20,35%	HIP	231	Side	GLN	227	Main	18,70%
**GLY**	221	Main	SER	218	Main	19,95%	GLN	146	Main	GLY	219	Main	18,50%
**VAL**	130	Main	TYR	294	Main	19,85%	GLY	221	Main	SER	218	Main	18,35%
**ALA**	252	Main	ASP	248	Main	19,50%	THR	210	Main	GLH	232	Main	18,15%
**THR**	155	Main	TRP	151	Main	19,15%	SER	154	Side	VAL	289	Main	17,85%
**SER**	336	Side	ALA	337	Main	18,95%	ASN	307	Main	GLY	317	Main	17,70%
**TRP**	151	Side	GLY	187	Main	18,55%	TRP	151	Side	GLY	187	Main	17,40%
**CYX**	188	Main	GLN	223	Side	18,45%	TRP	132	Side	LEU	254	Main	17,10%
**VAL**	201	Main	PHE	197	Main	18,45%	SER	298	Main	ASN	295	Side	16,90%
**GLH**	343	Side	ASP	239	Side	18,20%	TYR	294	Main	VAL	130	Main	16,80%
**GLN**	223	Side	VAL	179	Main	18,10%	SER	149	Side	TYR	216	Side	16,40%
**LYS**	142	Side	GLH	175	Side	17,85%	ASN	158	Side	PRO	259	Main	15,95%
**GLU**	315	Main	TYR	318	Main	17,75%	ALA	266	Main	ASH	286	Side	15,95%
**GLY**	157	Main	PHE	153	Main	17,40%	LEU	192	Main	ASH	185	Side	15,85%

**LYS**	142	Side	GLU	211	Side	17,20%	GLN	162	Side	PRO	259	Main	15,45%
**LEU**	192	Main	ASH	185	Side	16,65%	LYS	142	Side	GLU	160	Side	15,30%
**ASN**	158	Side	PRO	259	Main	16,65%	SER	308	Side	GLN	144	Side	15,20%
**GLN**	146	Main	GLY	219	Main	16,65%	LYS	142	Side	GLU	211	Side	14,75%
**LEU**	290	Main	LYS	306	Main	16,55%	PHE	186	Main	GLY	190	Main	14,65%
**SER**	298	Main	ASN	295	Side	16,10%	GLU	315	Main	TYR	318	Main	14,35%
**GLN**	162	Side	PRO	259	Main	15,60%	LYS	306	Side	THR	139	Main	14,30%
**ARG**	133	Main	ASP	131	Side	15,35%	VAL	201	Main	PHE	197	Main	13,75%
**TRP**	132	Side	LEU	254	Main	15,35%	SER	336	Side	ALA	337	Main	13,55%
**ASH**	185	Main	ASP	182	Side	15,05%	GLH	232	Main	SER	213	Side	13,50%
**GLH**	134	Side	ASP	131	Side	14,75%	ALA	255	Main	ALA	251	Main	13,50%
**ILE**	319	Main	ILE	305	Main	14,50%	GLN	223	Side	VAL	179	Main	13,15%
**ASP**	265	Main	CYX	328	Main	14,05%	ILE	200	Main	ALA	196	Main	13,05%
**TRP**	199	Main	ASN	195	Main	13,45%	TYR	214	Main	THR	210	Main	12,50%
**PHE**	269	Main	ALA	266	Main	13,40%	ALA	196	Main	LEU	192	Main	12,20%
**ALA**	255	Main	ALA	251	Main	13,30%	SER	203	Main	TRP	199	Main	12,15%
**HIP**	231	Side	GLN	227	Main	13,20%	ASN	202	Side	ASN	198	Main	11,75%
**LYS**	306	Side	PRO	140	Main	13,15%	ASP	182	Main	LEU	178	Main	11,70%
**LYS**	323	Side	ASP	248	Side	12,65%	GLY	157	Main	PHE	153	Main	11,60%
**ASN**	198	Main	ASH	194	Main	12,40%	ILE	319	Main	ILE	305	Main	11,30%
**ILE**	200	Main	ALA	196	Main	12,25%	ASH	185	Main	ASP	182	Side	11,25%
**SER**	172	Side	GLU	211	Side	12,15%	LYS	306	Side	PRO	140	Main	11,15%
**GLH**	232	Main	SER	213	Side	11,75%	ASN	207	Main	ASN	204	Main	11,05%
**TYR**	253	Main	ALA	249	Main	11,35%	LYS	135	Main	ASP	131	Main	11,00%
**GLU**	222	Main	ASN	220	Side	11,35%	GLY	145	Main	ASH	143	Side	10,75%
**ASN**	207	Main	ASN	204	Main	11,05%	GLH	256	Side	ALA	252	Main	10,70%
**ASN**	202	Side	ASN	198	Main	10,95%	TYR	253	Main	ALA	249	Main	10,60%
**TYR**	346	Main	GLH	343	Main	10,90%	ASP	248	Main	ASP	246	Side	10,05%
**ALA**	137	Main	TRP	132	Main	10,80%	ASN	257	Side	TYR	253	Main	9,85%
**LEU**	345	Main	PRO	342	Main	10,80%	ARG	133	Main	ASP	131	Side	9,80%
**LYS**	306	Side	THR	139	Main	10,00%	SER	335	Side	ASP	242	Side	9,80%
**ASP**	248	Main	ASP	246	Side	9,95%	ALA	261	Main	SER	335	Main	9,75%
**GLY**	189	Main	PHE	186	Main	9,90%	LYS	323	Side	ASP	248	Side	9,65%
**GLY**	317	Main	TRP	313	Main	9,80%	PHE	269	Main	ALA	266	Main	9,50%
**ASP**	182	Main	LEU	178	Main	9,75%	GLU	222	Main	ASN	220	Side	9,45%
**HIP**	287	Main	VAL	264	Main	9,70%	LYS	142	Side	GLH	175	Side	9,35%
**TYR**	302	Main	ASN	295	Main	9,60%	TYR	302	Main	ASN	295	Main	9,30%
**TYR**	214	Main	THR	210	Main	9,35%	ASN	198	Main	ASH	194	Main	9,25%
**GLN**	348	Side	ASN	344	Main	9,20%	PHE	347	Main	ASN	344	Main	9,20%
**GLY**	206	Main	ILE	200	Main	8,95%	ASP	265	Main	CYX	328	Main	8,80%
**ASN**	207	Side	ASN	204	Main	8,40%	THR	281	Main	SER	268	Side	8,55%
**ASH**	286	Side	ASP	265	Side	8,25%	TYR	346	Main	GLH	343	Main	8,40%
**ASN**	229	Main	GLN	227	Side	8,10%	ARG	133	Side	ASP	131	Side	8,10%
**SER**	203	Main	TRP	199	Main	7,95%	SER	154	Side	CYM	150	Main	7,85%
**VAL**	179	Main	GLH	175	Main	7,85%	TYR	318	Main	GLU	315	Main	7,60%
**SER**	154	Side	ILE	262	Main	7,85%	ASN	344	Side	ASN	257	Side	7,50%
**GLH**	343	Side	HIP	240	Main	7,80%	GLY	189	Main	PHE	186	Main	7,40%

**GLN**	245	Side	ASN	331	Side	7,70%	GLN	227	Side	ASN	229	Main	7,35%
**TYR**	318	Main	GLU	315	Main	7,30%	SER	310	Main	LYS	142	Main	7,30%
**SER**	310	Side	TRP	309	Main	7,05%	GLY	317	Main	TRP	313	Main	7,30%
**GLN**	164	Side	ASN	168	Main	6,90%	ALA	251	Main	GLU	247	Main	7,20%
**GLY**	288	Main	CYM	150	Side	6,90%	ASN	195	Side	ASH	185	Side	7,15%
**PHE**	186	Main	GLY	190	Main	6,85%	GLN	348	Side	ASN	344	Main	7,15%
**LYS**	323	Side	ASH	296	Side	6,80%	GLY	206	Main	ILE	200	Main	7,10%
**SER**	335	Side	ALA	261	Main	6,75%	GLN	348	Side	GLH	256	Main	7,05%
**THR**	281	Main	SER	268	Side	6,55%	GLN	332	Side	ASN	331	Side	7,00%
**LYS**	142	Side	SER	172	Side	6,50%	LYS	142	Side	SER	172	Side	6,85%
**ASN**	229	Side	GLN	227	Side	6,45%	GLN	245	Side	ASN	331	Side	6,80%
**ALA**	261	Main	SER	335	Main	6,35%	ASN	207	Side	ASN	204	Main	6,65%
**ASN**	331	Side	GLU	247	Side	6,35%	VAL	179	Main	GLH	175	Main	6,50%
**THR**	325	Side	GLH	322	Side	6,25%	SER	218	Main	GLN	176	Side	6,45%
**TYR**	346	Side	ASN	257	Side	6,25%	SER	310	Side	TRP	309	Main	6,40%
**GLY**	145	Main	ASH	143	Side	6,00%	GLN	348	Side	ASN	257	Side	6,15%
**GLN**	332	Side	LEU	329	Main	5,85%	TYR	216	Main	GLU	211	Side	5,75%
**MET**	330	Main	GLN	327	Main	5,80%	HIP	231	Side	SER	213	Main	5,55%
**ASN**	295	Side	TYR	302	Side	5,30%	LEU	345	Main	GLH	343	Side	5,55%
**ALA**	251	Main	GLU	247	Main	5,20%	LYS	323	Side	ASH	296	Side	5,35%
**ASN**	257	Side	TYR	253	Main	5,05%	SER	180	Side	GLN	176	Main	5,35%
							ASN	295	Side	TYR	302	Side	5,10%
							ALA	333	Main	MET	330	Main	5,05%
							GLH	134	Main	ASP	131	Side	5,05%
							GLN	146	Side	ASN	220	Main	5,00%

Table S4b: Relative occurrence of hydrogen bonds within the catalytic domain. Four respective MD simulations at pH 4 (pH4_3 and pH4_4, see Table S4a for pH4_1 and pH4_2) were carried out. `Main' refers to interactions of the backbone. 'Side' indicates interactions of side chains. Occurrences below 5% were left out for clarity.

pH4_3							pH4_4						
donor			acceptor			occupancy	donor			acceptor			occupancy
ASH	185	Side	ASP	182	Side	77,36%	ASH	222	Side	ASP	182	Side	82,91%
THR	267	Side	ASP	265	Side	73,16%	THR	304	Side	ASP	265	Side	78,21%
THR	155	Side	TRP	151	Main	72,96%	TYR	309	Side	GLY	275	Main	77,21%
ASH	194	Side	ASP	242	Side	70,36%	ARG	357	Side	GLU	315	Side	76,61%
TYR	272	Side	GLY	275	Main	69,07%	THR	192	Side	TRP	151	Main	72,51%
TRP	303	Main	ILE	321	Main	68,62%	TRP	340	Main	ILE	321	Main	67,42%
ARG	320	Side	GLU	315	Side	64,77%	ASH	231	Side	ASP	242	Side	64,32%
GLH	283	Main	ASP	265	Side	61,02%	ALA	303	Main	ASH	286	Side	60,57%
LYS	306	Main	LEU	290	Main	60,77%	GLH	320	Main	ASP	265	Side	56,92%
THR	139	Side	GLU	160	Side	58,47%	LYS	343	Main	LEU	290	Main	56,57%
ARG	133	Side	ASP	131	Side	56,82%	ILE	193	Main	ALA	152	Main	56,42%
PHE	197	Main	MET	193	Main	56,47%	PHE	234	Main	MET	193	Main	56,12%
VAL	241	Main	SER	336	Main	56,22%	VAL	278	Main	SER	336	Main	55,17%
TYR	216	Side	GLN	144	Main	55,47%	SER	211	Side	TYR	214	Main	53,82%
SER	174	Main	PHE	209	Main	55,37%	ARG	170	Side	ASP	131	Side	52,77%

GLH	175	Side	GLU	211	Side	53,97%	TYR	253	Side	GLN	144	Main	52,32%
SER	174	Side	TYR	214	Main	53,12%	GLN	199	Main	ASN	158	Main	52,22%
HIP	287	Side	ASN	307	Side	52,77%	SER	211	Main	PHE	209	Main	51,42%
ILE	156	Main	ALA	152	Main	51,72%	TYR	331	Side	PRO	127	Main	50,62%
SER	298	Side	PRO	300	Main	51,27%	ASH	333	Main	ALA	128	Main	50,27%
ALA	337	Main	ASN	158	Side	51,22%	CYX	317	Main	GLN	327	Side	50,22%
LEU	173	Main	GLU	160	Side	49,08%	HIP	324	Side	ASN	307	Side	50,12%
VAL	217	Main	GLN	176	Side	48,28%	ASH	323	Side	ASP	265	Side	49,08%
GLN	144	Side	CYX	147	Main	48,13%	GLY	312	Main	GLU	315	Side	48,53%
SER	282	Side	CYX	280	Main	45,38%	SER	335	Side	PRO	300	Main	48,48%
ASH	296	Main	ALA	128	Main	45,28%	LEU	328	Main	LEU	260	Main	48,23%
GLN	162	Main	ASN	158	Main	44,13%	TRP	169	Main	VAL	292	Main	47,23%
CYX	280	Main	GLN	327	Side	43,88%	SER	250	Side	THR	210	Side	46,58%
ASN	168	Side	TRP	163	Main	43,83%	VAL	254	Main	GLN	176	Side	46,28%
GLU	160	Main	ILE	156	Main	43,68%	ILE	274	Main	GLY	206	Main	45,53%
GLU	211	Main	SER	172	Main	42,83%	VAL	326	Main	ILE	262	Main	44,33%
LEU	291	Main	LEU	260	Main	42,38%	ASN	205	Side	TRP	163	Main	44,28%
ASH	286	Main	VAL	264	Main	42,18%	GLU	197	Main	ILE	156	Main	44,23%
THR	238	Main	VAL	338	Main	42,03%	LYS	360	Main	PRO	301	Main	43,98%
SER	213	Side	THR	210	Side	41,83%	THR	275	Main	VAL	338	Main	43,48%
GLY	275	Main	GLU	315	Side	41,58%	ALA	374	Main	ASN	158	Side	42,63%
ILE	250	Main	ASP	246	Main	40,58%	SER	255	Side	GLU	222	Main	41,93%
LYS	323	Main	PRO	301	Main	40,48%	GLH	359	Main	LEU	277	Main	41,93%
SER	218	Side	GLU	222	Main	40,48%	GLN	181	Side	CYX	147	Main	41,83%
ILE	237	Main	GLY	206	Main	39,93%	VAL	202	Main	GLY	161	Main	41,68%
GLH	322	Main	LEU	277	Main	39,73%	GLY	271	Main	VAL	208	Main	41,43%
GLY	234	Main	VAL	208	Main	39,53%	ASN	195	Side	SER	336	Side	39,18%
SER	154	Main	CYM	150	Main	39,43%	ILE	287	Main	ASP	246	Main	38,73%
GLY	324	Main	GLU	247	Side	38,43%	LEU	291	Main	ILE	250	Main	38,68%
SER	180	Main	GLN	176	Main	37,93%	THR	176	Side	GLU	160	Side	37,98%
ALA	266	Main	ASH	286	Side	37,88%	SER	345	Side	GLN	144	Side	37,38%
VAL	289	Main	ILE	262	Main	37,28%	VAL	301	Main	HIP	287	Main	36,98%
VAL	264	Main	HIP	287	Main	36,98%	ASH	323	Main	VAL	264	Main	36,93%
ASN	202	Main	ASN	198	Main	36,93%	ASN	239	Main	ASN	198	Main	36,13%
THR	267	Main	ASP	265	Side	36,08%	GLN	199	Side	ASN	257	Main	35,33%
TRP	303	Side	GLU	247	Side	35,38%	LEU	210	Main	GLU	160	Side	34,18%
LEU	254	Main	ILE	250	Main	35,08%	ILE	341	Main	GLY	293	Main	34,13%
ILE	305	Main	ILE	319	Main	33,68%	SER	191	Main	CYM	150	Main	34,03%
ILE	304	Main	GLY	293	Main	32,63%	GLN	213	Side	SER	218	Side	33,73%
ASN	158	Side	PRO	259	Main	32,58%	SER	217	Main	GLN	176	Main	33,53%
TRP	132	Main	VAL	292	Main	32,23%	TRP	200	Main	ILE	159	Main	32,63%
SER	154	Side	VAL	289	Main	32,13%	TRP	340	Side	GLU	247	Side	32,48%
SER	335	Main	ALA	261	Main	31,18%	GLU	248	Main	SER	172	Main	32,33%
VAL	292	Main	ILE	304	Main	31,13%	ASN	195	Side	PRO	259	Main	32,28%
SER	180	Side	PRO	224	Main	31,13%	GLY	361	Main	GLU	247	Side	31,53%
SER	336	Side	ALA	337	Main	31,03%	SER	319	Side	CYX	280	Main	30,68%
ASN	257	Main	TYR	253	Main	30,28%	ASN	241	Side	ASN	207	Main	30,48%

ASN	273	Side	TYR	272	Main	28,74%	GLN	181	Main	SER	308	Main	30,33%
ASN	204	Side	ASN	207	Main	28,59%	ILE	342	Main	ILE	319	Main	30,23%
GLN	284	Main	ASP	265	Side	28,54%	ASN	294	Main	TYR	253	Main	29,74%
GLN	176	Side	SER	218	Side	28,34%	LEU	280	Main	VAL	334	Main	29,19%
VAL	338	Main	ASP	239	Main	27,99%	VAL	167	Main	TYR	294	Main	28,49%
ASN	331	Main	ASN	326	Side	27,99%	SER	209	Side	GLU	160	Side	28,49%
LEU	277	Main	ARG	320	Main	27,84%	ASN	368	Side	GLN	245	Main	28,44%
GLN	144	Main	SER	308	Main	27,74%	LYS	179	Side	GLU	211	Side	27,39%
ASN	158	Side	SER	336	Side	27,69%	VAL	245	Main	ALA	235	Main	27,34%
SER	308	Side	GLN	144	Side	27,69%	SER	372	Main	ALA	261	Main	27,09%
THR	155	Main	TRP	151	Main	27,19%	VAL	375	Main	ASP	239	Main	26,19%
TYR	294	Main	VAL	130	Main	26,94%	ASN	332	Main	TYR	302	Main	25,59%
SER	335	Side	ASP	242	Side	26,19%	PHE	306	Main	ALA	266	Main	25,59%
HIP	287	Main	VAL	264	Main	26,09%	THR	304	Main	ASP	265	Side	25,19%
HIP	287	Side	CYM	150	Side	25,29%	ILE	221	Main	ASP	182	Side	24,99%
GLN	327	Side	THR	325	Side	25,09%	GLN	213	Side	SER	180	Side	23,49%
TRP	163	Main	ILE	159	Main	23,99%	TYR	253	Main	GLU	211	Side	22,59%
CYX	188	Main	GLN	223	Side	23,89%	VAL	329	Main	ILE	304	Main	22,24%
ASN	326	Main	GLU	247	Side	23,79%	LEU	314	Main	ARG	320	Main	21,94%
GLN	146	Main	GLY	219	Main	23,74%	SER	191	Side	CYM	150	Main	21,44%
GLN	176	Side	SER	180	Side	23,69%	ALA	289	Main	ASP	248	Main	21,34%
VAL	208	Main	ALA	235	Main	23,29%	PHE	223	Main	GLY	190	Main	21,14%
VAL	165	Main	GLY	161	Main	23,19%	GLY	377	Main	ALA	236	Main	21,04%
LEU	260	Main	LEU	291	Main	22,54%	CYX	225	Main	GLN	223	Side	20,94%
LYS	142	Side	GLU	160	Side	22,39%	TYR	331	Main	VAL	130	Main	20,84%
ILE	184	Main	ASP	182	Side	22,29%	GLY	354	Main	TRP	313	Main	20,64%
PHE	186	Main	GLY	190	Main	22,29%	SER	372	Side	ASP	242	Side	20,59%
ALA	255	Main	ALA	251	Main	21,89%	GLU	352	Main	TYR	318	Main	20,39%
ASN	307	Main	GLY	317	Main	21,09%	ASN	344	Main	GLY	317	Main	20,29%
LYS	306	Side	PRO	140	Main	20,59%	SER	373	Main	VAL	241	Main	20,24%
GLY	221	Main	SER	218	Main	20,39%	SER	186	Side	TYR	216	Side	20,04%
LEU	243	Main	VAL	334	Main	20,24%	ASP	302	Main	CYX	328	Main	19,94%
ASN	295	Main	TYR	302	Main	19,79%	ALA	174	Main	TRP	132	Main	19,49%
TRP	199	Main	ASN	195	Main	19,49%	ASN	310	Side	TYR	272	Main	19,24%
HIP	231	Side	GLN	227	Main	19,24%	HIP	268	Side	GLN	227	Main	19,09%
ALA	137	Main	TRP	132	Main	18,89%	SER	217	Side	GLN	176	Main	18,89%
SER	154	Side	ILE	262	Main	18,84%	TRP	188	Side	GLY	187	Main	18,84%
SER	336	Main	VAL	241	Main	18,64%	GLN	321	Main	ASP	265	Side	18,44%
ALA	252	Main	ASP	248	Main	18,59%	TYR	290	Main	ALA	249	Main	18,39%
ASN	257	Side	GLN	348	Side	18,54%	TRP	236	Main	ASN	195	Main	18,34%
LEU	192	Main	ASH	185	Side	18,39%	LYS	343	Side	THR	139	Main	18,19%
GLY	340	Main	ALA	236	Main	18,19%	GLN	364	Side	THR	325	Side	18,04%
THR	210	Main	GLH	232	Main	18,19%	ASN	363	Main	GLU	247	Side	17,94%
ALA	196	Main	LEU	192	Main	18,04%	SER	191	Side	VAL	289	Main	17,29%
TYR	253	Main	ALA	249	Main	17,79%	GLN	183	Main	GLY	219	Main	16,94%
ILE	200	Main	ALA	196	Main	16,99%	TRP	350	Side	PHE	269	Main	16,79%
VAL	130	Main	TYR	294	Main	16,49%	GLY	258	Main	SER	218	Main	16,64%

TRP	151	Side	GLY	187	Main	16,04%	ILE	356	Main	ILE	305	Main	16,54%
VAL	201	Main	PHE	197	Main	15,99%	THR	247	Main	GLH	232	Main	16,49%
TRP	313	Side	PHE	269	Main	15,89%	LEU	229	Main	ASH	185	Side	16,44%
GLN	162	Side	ASN	257	Main	15,79%	ARG	170	Main	ASP	131	Side	16,44%
SER	149	Side	TYR	216	Side	15,64%	THR	192	Main	TRP	151	Main	15,99%
SER	298	Main	ASN	295	Side	14,69%	HIP	324	Side	CYM	150	Side	15,29%
PHE	347	Main	ASN	344	Main	14,19%	LEU	297	Main	LEU	291	Main	15,24%
GLU	315	Main	TYR	318	Main	14,04%	VAL	238	Main	PHE	197	Main	14,99%
LEU	290	Main	LYS	306	Main	14,04%	ASN	363	Side	GLU	247	Side	14,44%
GLH	232	Main	SER	213	Side	13,74%	ILE	299	Main	VAL	289	Main	13,99%
LYS	142	Side	GLU	211	Side	13,74%	GLN	260	Side	VAL	179	Main	13,89%
ASH	185	Main	ASP	182	Side	13,64%	ALA	292	Main	ALA	251	Main	13,59%
LYS	323	Side	ASP	248	Side	13,49%	SER	191	Side	ILE	262	Main	13,24%
GLN	348	Side	ASN	257	Side	13,24%	SER	217	Side	PRO	224	Main	13,19%
GLN	223	Side	VAL	179	Main	13,19%	TYR	251	Main	THR	210	Main	13,19%
TYR	214	Main	THR	210	Main	12,59%	SER	335	Main	ASN	295	Side	13,09%
ARG	133	Main	ASP	131	Side	12,54%	GLH	269	Main	SER	213	Side	12,99%
ASN	195	Side	ASH	185	Side	11,49%	TRP	169	Side	LEU	254	Main	12,99%
GLY	145	Main	ASH	143	Side	11,44%	GLY	182	Main	ASH	143	Side	12,54%
TRP	132	Side	LEU	254	Main	11,34%	GLN	369	Main	LEU	329	Main	12,44%
ASN	207	Main	ASN	204	Main	11,19%	ASN	239	Side	ASN	198	Main	12,14%
LYS	142	Side	SER	172	Side	10,79%	ILE	237	Main	ALA	196	Main	12,04%
TYR	216	Main	GLU	211	Side	10,74%	ASH	222	Main	ASP	182	Side	11,69%
THR	325	Side	GLH	322	Side	10,69%	MET	367	Main	GLN	327	Main	11,69%
GLN	162	Side	PRO	259	Main	10,54%	SER	347	Side	TRP	309	Main	11,59%
ASN	198	Main	ASH	194	Main	10,49%	ASN	244	Main	ASN	204	Main	11,44%
GLY	157	Main	PHE	153	Main	10,34%	ALA	233	Main	LEU	192	Main	11,19%
TYR	302	Main	ASN	295	Main	10,29%	HIP	324	Main	VAL	264	Main	10,84%
GLH	175	Side	GLU	160	Side	10,24%	SER	240	Main	TRP	199	Main	10,54%
ASN	202	Side	ASN	198	Main	10,24%	PHE	384	Main	ASN	344	Main	10,19%
PHE	269	Main	ALA	266	Main	10,14%	GLN	264	Side	ASN	229	Main	10,00%
GLY	206	Main	ILE	200	Main	9,90%	THR	318	Main	SER	268	Side	9,75%
LYS	306	Side	THR	139	Main	9,70%	TYR	383	Main	GLH	343	Main	9,60%
GLY	317	Main	TRP	313	Main	9,50%	TYR	339	Main	ASN	295	Main	9,55%
LYS	142	Side	GLH	175	Side	9,15%	GLN	369	Side	ASN	331	Side	9,55%
ASN	344	Side	ASN	257	Side	8,85%	ALA	288	Main	GLU	247	Main	9,50%
SER	203	Main	TRP	199	Main	8,45%	ASP	219	Main	LEU	178	Main	9,50%
GLU	222	Main	ASN	220	Side	8,40%	ASN	235	Main	ASH	194	Main	9,45%
ASP	248	Main	ASP	246	Side	8,40%	ASN	381	Side	GLY	167	Main	9,35%
ASN	257	Side	TYR	253	Main	8,35%	LYS	360	Side	ASH	296	Side	9,20%
GLN	227	Side	ASN	229	Main	8,35%	GLY	194	Main	PHE	153	Main	9,15%
ILE	319	Main	ILE	305	Main	8,05%	ASN	368	Main	ASN	326	Side	8,55%
ASP	265	Main	CYX	328	Main	8,00%	ASP	285	Main	ASP	246	Side	8,15%
LYS	323	Side	ASH	296	Side	7,85%	VAL	216	Main	GLH	175	Main	7,90%
VAL	179	Main	GLH	175	Main	7,60%	GLY	243	Main	ILE	200	Main	7,85%
TYR	318	Main	GLU	315	Main	7,30%	LEU	327	Main	LYS	306	Main	6,95%
GLY	189	Main	PHE	186	Main	7,25%	GLU	259	Main	ASN	220	Side	6,90%

ASN	331	Side	GLU	247	Side	7,15%	ALA	298	Main	SER	335	Main	6,75%
ASP	182	Main	LEU	178	Main	6,95%	TYR	309	Main	PHE	269	Main	6,60%
HIP	240	Side	ASP	242	Side	6,85%	SER	373	Side	ALA	337	Main	6,50%
MET	330	Main	GLN	327	Main	6,85%	ASN	244	Side	ASN	204	Main	6,40%
SER	310	Side	TRP	309	Main	6,80%	THR	362	Side	GLH	322	Side	6,30%
ALA	333	Main	MET	330	Main	6,80%	LYS	172	Main	ASP	131	Main	6,10%
SER	172	Side	GLU	160	Side	6,65%	ASN	294	Side	TYR	253	Main	6,10%
THR	281	Main	SER	268	Side	6,55%	ASN	232	Side	ASH	185	Side	5,95%
ASN	207	Side	ASN	204	Main	5,95%	GLY	226	Main	PHE	186	Main	5,95%
GLH	256	Side	GLN	348	Side	5,95%	ASN	266	Side	GLN	227	Side	5,85%
CYX	181	Main	MET	177	Main	5,85%	LYS	179	Side	GLH	175	Side	5,75%
ASN	295	Side	TYR	302	Side	5,50%	SER	255	Side	GLN	176	Side	5,75%
SER	218	Main	GLN	176	Side	5,30%	SER	372	Side	ALA	261	Main	5,70%
GLN	245	Side	ASN	331	Side	5,30%	ASN	344	Side	TRP	313	Side	5,45%
ASN	344	Side	GLH	256	Main	5,25%	ASN	266	Main	GLN	227	Side	5,35%
SER	180	Side	GLN	176	Main	5,10%	GLH	293	Side	GLN	348	Main	5,35%
GLN	348	Side	GLN	348	Side	5,10%	LEU	382	Main	ALA	166	Main	5,30%
							SER	255	Main	GLN	176	Side	5,25%
							ASN	381	Side	ASN	168	Side	5,15%
							HIP	268	Side	SER	213	Main	5,10%

Table S5a: Relative occurrence of hydrogen bonds within the catalytic domain. Four respective MD simulations at pH 8 (pH8_1 and pH8_2, see Table S5b for pH8_3 and pH8_4) were carried out. `Main' refers to interactions of the backbone. 'Side' indicates interactions of side chains. Occurrences below 5% were left out for clarity.

pH8_1							pH8_2						
donor			acceptor			occupancy	donor			acceptor			occupancy
THR	267	Side	ASP	265	Side	73,80%	THR	267	Side	ASP	265	Side	76,40%
THR	155	Side	TRP	151	Main	72,80%	TYR	272	Side	GLY	275	Main	73,35%
TYR	272	Side	GLY	275	Main	69,25%	THR	155	Side	TRP	151	Main	72,40%
TRP	303	Main	ILE	321	Main	67,35%	TRP	303	Main	ILE	321	Main	67,95%
THR	139	Side	GLU	160	Side	66,75%	LYS	306	Main	LEU	290	Main	63,10%
ILE	156	Main	ALA	152	Main	63,70%	ARG	320	Side	GLU	315	Side	62,70%
SER	218	Side	GLU	222	Main	63,65%	SER	218	Side	GLU	222	Main	60,05%
GLU	283	Main	ASP	265	Side	61,80%	ILE	156	Main	ALA	152	Main	59,80%
ASN	195	Side	ASP	185	Side	59,85%	ARG	133	Side	ASP	131	Side	59,15%
LYS	306	Main	LEU	290	Main	59,45%	THR	139	Side	GLU	160	Side	58,05%
ARG	320	Side	GLU	315	Side	58,95%	GLU	283	Main	ASP	265	Side	57,30%
PHE	197	Main	MET	193	Main	58,00%	VAL	217	Main	GLN	176	Side	55,50%
HIP	287	Side	ASN	307	Side	57,95%	LYS	323	Side	ASP	296	Side	54,90%
LEU	173	Main	GLU	160	Side	57,65%	HIP	287	Side	ASN	307	Side	54,30%
ALA	266	Main	ASP	286	Side	57,65%	PHE	197	Main	MET	193	Main	53,75%
SER	335	Side	ASP	194	Side	56,80%	TYR	216	Side	GLN	144	Main	53,15%
SER	336	Side	ALA	337	Main	55,25%	VAL	241	Main	SER	336	Main	52,70%
VAL	217	Main	GLN	176	Side	54,25%	LEU	173	Main	GLU	160	Side	52,60%
ARG	133	Side	ASP	131	Side	54,10%	ALA	337	Main	ASN	158	Side	52,10%
CYX	280	Main	GLN	327	Side	54,05%	ASP	296	Main	ALA	128	Main	50,70%

ALA	337	Main	ASN	158	Side	53,65%	SER	336	Side	ALA	337	Main	50,65%
GLU	160	Main	ILE	156	Main	52,05%	SER	298	Side	PRO	300	Main	50,40%
LYS	323	Side	ASP	296	Side	50,95%	SER	335	Side	ASP	242	Side	50,20%
VAL	241	Main	SER	336	Main	50,70%	GLN	162	Main	ASN	158	Main	49,45%
GLN	162	Main	ASN	158	Main	49,25%	ALA	266	Main	ASP	286	Side	49,20%
SER	282	Side	CYX	280	Main	47,80%	GLU	160	Main	ILE	156	Main	46,60%
GLU	322	Main	LEU	277	Main	47,40%	LYS	142	Side	GLU	175	Side	46,30%
GLN	144	Side	CYX	147	Main	46,90%	GLN	144	Side	CYX	147	Main	45,65%
ASN	202	Main	ASN	198	Main	46,70%	GLU	322	Main	LEU	277	Main	45,15%
ASP	296	Main	ALA	128	Main	46,20%	ASN	158	Side	SER	336	Side	44,85%
ILE	250	Main	ASP	246	Main	45,65%	CYX	280	Main	GLN	327	Side	44,10%
TYR	216	Side	GLN	144	Main	45,10%	GLN	176	Side	SER	218	Side	43,90%
LYS	142	Side	GLU	175	Side	44,65%	ASN	168	Side	TRP	163	Main	43,60%
SER	154	Side	VAL	289	Main	43,90%	SER	282	Side	CYX	280	Main	43,25%
ASN	168	Side	TRP	163	Main	43,15%	SER	213	Side	THR	210	Side	43,00%
LEU	291	Main	LEU	260	Main	42,90%	VAL	289	Main	ILE	262	Main	42,20%
GLN	176	Side	SER	218	Side	42,70%	GLY	275	Main	GLU	315	Side	41,55%
SER	172	Side	GLU	160	Side	42,10%	ILE	237	Main	GLY	206	Main	41,55%
SER	298	Side	PRO	300	Main	41,20%	LEU	291	Main	LEU	260	Main	40,65%
VAL	289	Main	ILE	262	Main	41,15%	SER	335	Main	ALA	261	Main	40,60%
GLY	275	Main	GLU	315	Side	40,75%	ILE	250	Main	ASP	246	Main	39,95%
GLY	324	Main	GLU	247	Side	40,40%	GLY	324	Main	GLU	247	Side	38,25%
SER	154	Main	CYM	150	Main	40,00%	SER	308	Side	GLN	144	Side	38,15%
ASN	158	Side	SER	336	Side	39,55%	SER	174	Main	PHE	209	Main	37,85%
SER	213	Side	THR	210	Side	39,45%	SER	154	Side	VAL	289	Main	37,65%
ILE	237	Main	GLY	206	Main	39,10%	GLN	162	Side	PRO	259	Main	37,30%
ILE	305	Main	ILE	319	Main	36,85%	SER	336	Main	VAL	241	Main	36,05%
TRP	303	Side	GLU	247	Side	35,90%	SER	174	Side	TYR	214	Main	35,55%
VAL	264	Main	HIP	287	Main	35,25%	ILE	305	Main	ILE	319	Main	35,30%
SER	174	Main	PHE	209	Main	35,10%	SER	154	Main	CYM	150	Main	34,55%
SER	180	Main	GLN	176	Main	34,45%	THR	238	Main	VAL	338	Main	34,15%
ILE	304	Main	GLY	293	Main	33,95%	SER	180	Main	GLN	176	Main	34,05%
LEU	254	Main	ILE	250	Main	33,90%	ILE	304	Main	GLY	293	Main	33,90%
ILE	200	Main	ALA	196	Main	33,45%	LYS	323	Main	PRO	301	Main	33,45%
SER	180	Side	PRO	224	Main	33,35%	SER	172	Side	GLU	160	Side	33,35%
ILE	184	Main	ASP	182	Side	33,25%	LYS	142	Side	GLU	160	Side	33,15%
LYS	142	Side	GLU	160	Side	33,05%	TRP	132	Main	VAL	292	Main	32,80%
TRP	151	Side	GLY	187	Main	32,95%	GLU	211	Main	SER	172	Main	31,85%
SER	174	Side	TYR	214	Main	32,75%	VAL	264	Main	HIP	287	Main	31,75%
LYS	323	Main	PRO	301	Main	32,45%	GLN	176	Side	SER	180	Side	31,50%
GLN	146	Main	GLY	219	Main	32,40%	ASN	331	Main	ASN	326	Side	30,70%
TRP	199	Main	ASN	195	Main	31,65%	TRP	303	Side	GLU	247	Side	30,55%
GLN	176	Side	SER	180	Side	31,30%	GLY	234	Main	VAL	208	Main	29,65%
THR	325	Side	GLU	322	Side	31,05%	ASN	202	Main	ASN	198	Main	29,15%
CYX	188	Main	GLN	223	Side	30,35%	VAL	292	Main	ILE	304	Main	29,10%
VAL	292	Main	ILE	304	Main	29,80%	ASN	273	Side	TYR	272	Main	29,10%
ASN	331	Main	ASN	326	Side	29,75%	LEU	260	Main	LEU	291	Main	29,05%

SER	335	Main	ALA	261	Main	29,65%	GLN	146	Main	GLY	219	Main	28,35%
THR	238	Main	VAL	338	Main	29,35%	ILE	200	Main	ALA	196	Main	28,25%
ASP	286	Main	VAL	264	Main	27,70%	THR	267	Main	ASP	265	Side	27,70%
VAL	130	Main	TYR	294	Main	27,55%	ASN	204	Side	ASN	207	Main	27,45%
GLU	211	Main	SER	172	Main	27,10%	LEU	254	Main	ILE	250	Main	26,95%
THR	267	Main	ASP	265	Side	27,10%	VAL	130	Main	TYR	294	Main	26,75%
TRP	132	Main	VAL	292	Main	26,45%	ASN	307	Main	GLY	317	Main	26,15%
LYS	306	Side	PRO	140	Main	26,20%	GLN	284	Main	ASP	265	Side	25,85%
GLY	234	Main	VAL	208	Main	25,85%	VAL	338	Main	ASP	239	Main	25,85%
ASN	257	Main	TYR	253	Main	25,85%	LEU	243	Main	VAL	334	Main	25,25%
GLN	144	Main	SER	308	Main	25,55%	THR	210	Main	GLU	232	Main	25,10%
TYR	294	Main	VAL	130	Main	25,15%	VAL	208	Main	ALA	235	Main	24,80%
HIP	287	Side	CYM	150	Side	24,60%	ASP	286	Main	VAL	264	Main	24,80%
GLN	162	Side	PRO	259	Main	23,85%	CYX	188	Main	GLN	223	Side	24,20%
THR	210	Main	GLU	232	Main	23,40%	THR	325	Side	GLU	322	Side	23,80%
LEU	277	Main	ARG	320	Main	23,30%	TYR	294	Main	VAL	130	Main	23,60%
TYR	253	Main	ALA	249	Main	22,80%	ASN	295	Main	TYR	302	Main	22,45%
ASN	295	Main	TYR	302	Main	22,35%	GLN	144	Main	SER	308	Main	22,45%
TRP	163	Main	ILE	159	Main	22,30%	LEU	277	Main	ARG	320	Main	22,25%
ASN	307	Main	GLY	317	Main	21,15%	TRP	163	Main	ILE	159	Main	21,95%
GLN	284	Main	ASP	265	Side	20,60%	THR	155	Main	TRP	151	Main	21,75%
ASN	204	Side	ASN	207	Main	20,45%	HIP	287	Side	CYM	150	Side	21,50%
ASN	158	Side	PRO	259	Main	20,45%	GLU	232	Main	SER	213	Side	21,35%
VAL	165	Main	GLY	161	Main	20,40%	VAL	165	Main	GLY	161	Main	21,00%
GLU	232	Main	SER	213	Side	20,35%	HIP	287	Main	VAL	264	Main	20,70%
THR	155	Main	TRP	151	Main	19,75%	ASN	326	Main	GLU	247	Side	19,90%
ASN	326	Main	GLU	247	Side	19,70%	SER	203	Main	TRP	199	Main	19,90%
ALA	255	Main	ALA	251	Main	19,60%	TRP	199	Main	ASN	195	Main	19,75%
HIP	287	Main	VAL	264	Main	19,55%	LYS	306	Side	PRO	140	Main	19,45%
GLY	145	Main	ASP	143	Side	19,50%	ASN	198	Side	ASP	194	Main	18,65%
VAL	208	Main	ALA	235	Main	19,25%	ALA	255	Main	ALA	251	Main	18,50%
ALA	252	Main	ASP	248	Main	17,75%	GLU	315	Main	TYR	318	Main	18,40%
VAL	338	Main	ASP	239	Main	17,70%	ASN	195	Side	ASP	185	Side	17,15%
GLU	315	Main	TYR	318	Main	17,70%	GLY	221	Main	SER	218	Main	16,70%
ALA	196	Main	LEU	192	Main	17,40%	LEU	290	Main	LYS	306	Main	16,60%
ASP	185	Main	ASP	182	Side	16,65%	ALA	137	Main	TRP	132	Main	16,45%
VAL	201	Main	PHE	197	Main	16,60%	GLN	327	Side	THR	325	Side	16,35%
LEU	260	Main	LEU	291	Main	16,50%	GLY	189	Main	PHE	186	Main	16,10%
ASN	273	Side	TYR	272	Main	16,45%	ASN	158	Side	PRO	259	Main	15,70%
ALA	137	Main	TRP	132	Main	14,35%	GLY	317	Main	TRP	313	Main	15,70%
LEU	290	Main	LYS	306	Main	13,95%	SER	180	Side	GLN	176	Main	14,90%
TYR	214	Main	THR	210	Main	13,90%	ASP	182	Main	LEU	178	Main	14,80%
GLN	332	Side	ASN	331	Side	13,90%	TYR	253	Main	ALA	249	Main	14,55%
PHE	269	Main	ALA	266	Main	13,70%	GLY	145	Main	ASP	143	Side	14,55%
GLY	221	Main	SER	218	Main	13,55%	ALA	196	Main	LEU	192	Main	14,45%
SER	310	Main	LYS	142	Main	13,35%	ALA	252	Main	ASP	248	Main	14,40%
ARG	133	Main	ASP	131	Side	12,90%	ASP	265	Main	CYX	328	Main	14,10%

SER	310	Side	TRP	309	Main	12,85%	ASN	202	Side	ASN	198	Main	14,10%
ILE	319	Main	ILE	305	Main	12,65%	TRP	151	Side	GLY	187	Main	14,00%
SER	308	Side	GLN	144	Side	12,60%	TYR	346	Main	GLU	343	Main	13,40%
GLY	157	Main	PHE	153	Main	12,15%	ARG	133	Main	ASP	131	Side	13,05%
ASN	257	Side	TYR	253	Main	12,05%	TYR	214	Main	THR	210	Main	12,85%
GLU	222	Main	ASN	220	Side	11,85%	GLN	223	Side	VAL	179	Main	12,80%
GLN	327	Side	THR	325	Side	11,75%	ASN	257	Main	TYR	253	Main	12,60%
SER	154	Side	ILE	262	Main	11,55%	SER	180	Side	PRO	224	Main	12,50%
SER	172	Side	GLU	211	Side	10,75%	HIP	231	Side	GLN	227	Main	12,50%
SER	203	Main	TRP	199	Main	10,65%	ASN	207	Main	ASN	204	Main	12,45%
TYR	302	Main	ASN	295	Main	10,60%	TRP	132	Side	LEU	254	Main	11,75%
ASN	207	Main	ASN	204	Main	10,35%	TYR	302	Main	ASN	295	Main	11,35%
SER	298	Side	ASN	295	Side	10,20%	SER	218	Main	GLN	176	Side	11,35%
LEU	345	Main	GLU	343	Side	10,05%	VAL	179	Main	GLU	175	Main	11,30%
HIP	231	Side	GLN	227	Main	9,85%	SER	154	Side	ILE	262	Main	11,25%
ASP	248	Main	ASP	246	Side	9,85%	ASP	248	Main	ASP	246	Side	11,15%
ASN	331	Side	GLU	247	Side	9,85%	THR	281	Main	SER	268	Side	11,15%
LEU	192	Main	ASP	185	Side	9,75%	GLY	157	Main	PHE	153	Main	11,15%
ALA	333	Main	MET	330	Main	9,50%	TRP	313	Side	PHE	269	Main	10,75%
GLY	206	Main	ILE	200	Main	9,45%	SER	310	Side	TRP	309	Main	10,75%
GLY	189	Main	PHE	186	Main	9,45%	ILE	319	Main	ILE	305	Main	9,95%
SER	203	Side	ASN	202	Side	9,45%	ASP	185	Main	ASP	182	Side	9,75%
SER	298	Main	ASN	295	Side	9,40%	ILE	184	Main	ASP	182	Side	9,20%
GLY	317	Main	TRP	313	Main	8,90%	GLU	222	Main	ASN	220	Side	9,05%
TRP	132	Side	LEU	254	Main	8,75%	GLY	206	Main	ILE	200	Main	8,85%
ASN	207	Side	ASN	204	Main	8,70%	SER	172	Side	GLU	211	Side	8,70%
TYR	346	Main	GLU	343	Main	8,65%	ASN	207	Side	ASN	204	Main	8,60%
ASN	168	Side	GLY	340	Main	7,95%	VAL	201	Main	PHE	197	Main	8,50%
SER	336	Main	VAL	241	Main	7,80%	SER	149	Side	TYR	216	Side	8,35%
LEU	243	Main	VAL	334	Main	7,35%	MET	270	Main	ALA	266	Main	7,40%
TRP	313	Side	PHE	269	Main	6,90%	PHE	269	Main	ALA	266	Main	7,35%
PHE	347	Main	GLU	343	Main	6,80%	ALA	333	Main	MET	330	Main	7,10%
VAL	179	Main	GLU	175	Main	6,65%	GLY	340	Main	ALA	236	Main	6,75%
GLN	332	Side	GLN	245	Side	6,55%	HIP	231	Side	SER	213	Main	6,40%
ASN	202	Side	ASN	198	Main	6,35%	TYR	318	Main	GLU	315	Main	6,35%
GLN	164	Side	ASN	168	Main	6,25%	SER	298	Main	ASN	295	Side	6,20%
TYR	346	Side	GLY	234	Main	6,25%	ASN	295	Side	TYR	302	Side	6,20%
SER	180	Side	GLN	176	Main	6,15%	GLY	167	Main	GLN	164	Main	6,00%
GLN	245	Side	GLN	332	Side	6,00%	GLN	332	Side	LEU	329	Main	6,00%
MET	330	Main	GLN	327	Main	5,90%	ASN	257	Side	TYR	253	Main	5,75%
GLY	167	Main	GLN	164	Main	5,85%	ALA	251	Main	GLU	247	Main	5,65%
GLN	227	Side	ASN	229	Main	5,75%	ASN	198	Main	ASP	194	Main	5,60%
TYR	318	Main	GLU	315	Main	5,70%	MET	330	Main	GLN	327	Main	5,55%
ASP	265	Main	CYX	328	Main	5,55%	PHE	347	Main	ASN	344	Main	5,45%
GLN	162	Side	ASN	257	Main	5,45%	ASN	229	Main	GLN	227	Side	5,35%
GLN	223	Side	VAL	179	Main	5,45%	ASN	311	Side	ASP	316	Main	5,35%
GLN	348	Main	ASN	344	Main	5,30%	ASN	229	Side	GLN	227	Side	5,25%

SER	218	Main	GLN	176	Side	5,30%	SER	268	Side	THR	281	Main	5,25%
							LEU	178	Main	GLU	175	Main	5,20%
							GLN	227	Side	ASN	229	Main	5,10%

Table S5b: Relative occurrence of hydrogen bonds within the catalytic domain. Two respective MD simulations at pH 8 (pH8_3 and pH8_4, see Table S5a for pH8_1 and pH8_4) were carried out. `Main' refers to interactions of the backbone. 'Side' indicates interactions of side chains. Occurrences below 5% were left out for clarity.

pH_8_3							pH_8_4						
Found			388			hbonds,	Found			387			hbonds,
donor			acceptor			occupancy	donor			acceptor			occupancy
TYR	272	Side	GLY	275	Main	75,56%	TYR	272	Side	GLY	309	Main	73,21%
THR	267	Side	ASP	265	Side	74,11%	THR	155	Side	TRP	192	Main	72,36%
THR	155	Side	TRP	151	Main	71,71%	SER	335	Side	ASP	372	Side	72,21%
ARG	320	Side	GLU	315	Side	69,82%	ALA	266	Main	ASP	303	Side	71,86%
TRP	303	Main	ILE	321	Main	66,57%	SER	268	Side	ASP	305	Side	71,01%
SER	218	Side	GLU	222	Main	63,12%	TRP	303	Main	ILE	340	Main	70,56%
ILE	156	Main	ALA	152	Main	62,22%	LYS	306	Main	LEU	343	Main	64,32%
GLU	283	Main	ASP	265	Side	61,57%	ILE	156	Main	ALA	193	Main	64,07%
LEU	173	Main	GLU	160	Side	58,52%	ARG	320	Side	GLU	357	Side	58,02%
GLU	160	Main	ILE	156	Main	57,07%	SER	218	Side	GLU	255	Main	57,17%
THR	139	Side	GLU	160	Side	56,97%	HIP	287	Side	ASN	324	Side	56,12%
VAL	241	Main	SER	336	Main	53,32%	ARG	133	Side	ASP	170	Side	55,37%
TYR	216	Side	GLN	144	Main	52,72%	ASN	195	Side	ASP	232	Side	54,82%
ALA	337	Main	ASN	158	Side	52,02%	LEU	173	Main	GLU	210	Side	54,57%
HIP	287	Side	ASN	307	Side	52,02%	ASP	296	Main	ALA	333	Main	52,77%
ALA	266	Main	ASP	286	Side	51,57%	PHE	197	Main	MET	234	Main	52,57%
VAL	217	Main	GLN	176	Side	50,87%	LYS	323	Side	ASP	360	Side	52,17%
LYS	306	Main	LEU	290	Main	50,47%	TYR	216	Side	GLN	253	Main	50,82%
ASP	296	Main	ALA	128	Main	50,47%	GLN	162	Main	ASN	199	Main	50,32%
CYX	280	Main	GLN	327	Side	50,22%	SER	336	Side	ALA	373	Main	48,23%
GLN	162	Main	ASN	158	Main	49,68%	VAL	217	Main	GLN	254	Side	47,63%
LYS	323	Side	ASP	296	Side	47,88%	ASN	168	Side	TRP	205	Main	47,23%
LYS	142	Side	GLU	175	Side	47,33%	GLN	144	Side	CYX	181	Main	47,08%
GLN	144	Side	CYX	147	Main	46,68%	ALA	337	Main	ASN	374	Side	45,08%
GLY	275	Main	GLU	315	Side	46,23%	LEU	291	Main	LEU	328	Main	44,93%
SER	282	Side	CYX	280	Main	45,98%	THR	139	Side	GLU	176	Side	44,68%
SER	154	Side	VAL	289	Main	45,48%	LYS	142	Side	GLU	179	Side	44,58%
GLU	322	Main	LEU	277	Main	45,48%	LEU	277	Main	ARG	314	Main	44,33%
PHE	197	Main	MET	193	Main	44,68%	VAL	241	Main	SER	278	Main	42,73%
SER	336	Side	ALA	337	Main	44,28%	VAL	289	Main	ILE	326	Main	42,68%
LEU	291	Main	LEU	260	Main	43,43%	ILE	237	Main	GLY	274	Main	42,43%
VAL	289	Main	ILE	262	Main	43,33%	GLN	146	Main	GLY	183	Main	42,38%
ASN	168	Side	TRP	163	Main	43,18%	ILE	250	Main	ASP	287	Main	41,53%
SER	213	Side	THR	210	Side	43,13%	SER	154	Main	CYM	191	Main	40,73%
ILE	237	Main	GLY	206	Main	41,88%	SER	154	Side	VAL	191	Main	40,58%
SER	180	Main	GLN	176	Main	41,18%	GLN	176	Side	SER	213	Side	40,48%

GLN	176	Side	SER	218	Side	40,93%	VAL	264	Main	HIP	301	Main	40,23%
ARG	133	Side	ASP	131	Side	39,98%	SER	298	Side	PRO	335	Main	39,98%
SER	335	Main	ALA	261	Main	38,78%	ASN	158	Side	SER	195	Side	38,48%
ILE	250	Main	ASP	246	Main	38,78%	CYX	188	Main	GLN	225	Side	38,38%
SER	154	Main	CYM	150	Main	38,48%	ILE	305	Main	ILE	342	Main	38,18%
SER	298	Side	PRO	300	Main	37,03%	SER	213	Side	THR	250	Side	36,78%
GLN	146	Main	GLY	219	Main	36,88%	SER	174	Side	TYR	211	Main	36,58%
ASN	158	Side	SER	336	Side	36,73%	GLU	322	Main	LEU	359	Main	36,33%
ASN	202	Main	ASN	198	Main	36,63%	LYS	323	Main	PRO	360	Main	36,23%
SER	172	Side	GLU	160	Side	36,58%	ASN	202	Main	ASN	239	Main	35,43%
LYS	142	Side	GLU	160	Side	35,38%	ILE	304	Main	GLY	341	Main	35,43%
VAL	264	Main	HIP	287	Main	34,78%	TRP	151	Side	GLY	188	Main	35,38%
GLU	211	Main	SER	172	Main	34,68%	THR	238	Main	VAL	275	Main	34,78%
SER	174	Side	TYR	214	Main	34,43%	TRP	132	Main	VAL	169	Main	34,68%
SER	174	Main	PHE	209	Main	33,53%	VAL	292	Main	ILE	329	Main	34,43%
THR	238	Main	VAL	338	Main	31,98%	SER	335	Main	ALA	372	Main	34,38%
ILE	304	Main	GLY	293	Main	31,28%	LEU	254	Main	ILE	291	Main	34,33%
ASN	331	Main	ASN	326	Side	31,23%	SER	174	Main	PHE	211	Main	34,03%
LYS	323	Main	PRO	301	Main	30,88%	GLU	211	Main	SER	248	Main	33,53%
ASP	286	Main	VAL	264	Main	30,13%	GLU	160	Main	ILE	197	Main	33,53%
GLY	234	Main	VAL	208	Main	29,99%	ASP	185	Main	ASP	222	Side	33,18%
THR	267	Main	ASP	265	Side	29,79%	LYS	142	Side	GLU	179	Side	32,58%
GLY	324	Main	GLU	247	Side	29,44%	ILE	200	Main	ALA	237	Main	32,03%
LEU	254	Main	ILE	250	Main	29,34%	GLN	176	Side	SER	213	Side	31,98%
GLN	284	Main	ASP	265	Side	29,29%	SER	180	Main	GLN	217	Main	31,78%
SER	308	Side	GLN	144	Side	29,04%	ILE	184	Main	ASP	221	Side	31,38%
TRP	303	Side	GLU	247	Side	28,99%	SER	180	Side	PRO	217	Main	30,08%
ASN	257	Main	TYR	253	Main	28,94%	GLY	275	Main	GLU	312	Side	29,89%
ILE	305	Main	ILE	319	Main	28,64%	TRP	199	Main	ASN	236	Main	28,99%
GLN	176	Side	SER	180	Side	28,34%	GLY	234	Main	VAL	271	Main	28,74%
VAL	292	Main	ILE	304	Main	26,79%	VAL	130	Main	TYR	167	Main	27,94%
VAL	130	Main	TYR	294	Main	26,69%	HIP	287	Side	CYM	324	Side	27,09%
ASN	204	Side	ASN	207	Main	26,69%	THR	210	Main	GLU	247	Main	27,04%
THR	325	Side	GLU	322	Side	26,59%	ASN	295	Main	TYR	332	Main	27,04%
VAL	338	Main	ASP	239	Main	25,59%	VAL	208	Main	ALA	245	Main	26,74%
GLU	232	Main	SER	213	Side	24,64%	ASN	257	Main	TYR	294	Main	25,94%
LEU	243	Main	VAL	334	Main	24,29%	TRP	303	Side	GLU	340	Side	25,34%
TYR	294	Main	VAL	130	Main	23,79%	GLU	232	Main	SER	213	Side	25,29%
TRP	163	Main	ILE	159	Main	23,49%	PHE	347	Main	GLU	384	Main	24,89%
SER	335	Side	ASP	242	Side	23,39%	GLN	162	Side	PRO	199	Main	24,54%
LEU	277	Main	ARG	320	Main	23,19%	CYX	280	Main	GLN	317	Side	23,94%
VAL	165	Main	GLY	161	Main	23,19%	TYR	294	Main	VAL	331	Main	23,89%
LYS	306	Side	PRO	140	Main	22,99%	TYR	294	Side	PRO	331	Main	23,79%
ASN	307	Main	GLY	317	Main	22,84%	TRP	163	Main	ILE	200	Main	22,99%
ASN	295	Main	TYR	302	Main	22,84%	ASN	204	Side	ASN	241	Main	22,74%
HIP	287	Side	CYM	150	Side	22,84%	VAL	179	Main	GLU	216	Main	22,64%
ASN	195	Side	ASP	185	Side	22,79%	VAL	165	Main	GLY	202	Main	22,59%

THR	210	Main	GLU	232	Main	22,74%	VAL	338	Main	ASP	375	Main	22,49%
PHE	269	Main	ALA	266	Main	22,64%	THR	281	Side	ASP	318	Side	22,24%
SER	172	Side	GLU	211	Side	22,64%	GLN	144	Main	SER	181	Main	21,44%
GLU	315	Main	TYR	318	Main	22,54%	ASN	331	Main	ASN	368	Side	21,39%
HIP	287	Main	VAL	264	Main	22,49%	ALA	255	Main	ALA	292	Main	20,79%
GLN	223	Side	VAL	179	Main	22,49%	LEU	260	Main	LEU	297	Main	20,69%
TRP	132	Main	VAL	292	Main	22,19%	ALA	137	Main	TRP	174	Main	19,44%
ILE	200	Main	ALA	196	Main	21,79%	ASN	158	Side	PRO	195	Main	19,09%
VAL	208	Main	ALA	235	Main	21,29%	HIP	231	Side	GLN	268	Main	19,04%
GLN	144	Main	SER	308	Main	21,14%	GLY	145	Main	ASP	182	Side	18,89%
GLN	162	Side	PRO	259	Main	20,94%	GLY	324	Main	GLU	361	Side	18,79%
SER	335	Side	ASP	194	Side	20,79%	LYS	306	Side	PRO	343	Main	17,99%
ASN	158	Side	PRO	259	Main	19,99%	GLU	315	Main	TYR	352	Main	16,84%
SER	336	Main	VAL	241	Main	19,54%	ASN	326	Main	GLU	363	Side	16,74%
TYR	253	Main	ALA	249	Main	19,49%	ASN	307	Main	GLY	344	Main	16,74%
TRP	199	Main	ASN	195	Main	19,09%	TYR	253	Main	ALA	290	Main	16,29%
THR	155	Main	TRP	151	Main	18,64%	VAL	201	Main	PHE	238	Main	16,29%
GLN	327	Side	THR	325	Side	18,34%	ASN	198	Side	ASP	235	Main	15,84%
PHE	347	Main	GLU	343	Main	18,24%	THR	281	Main	SER	318	Side	15,59%
GLY	145	Main	ASP	143	Side	18,14%	ASP	182	Main	LEU	219	Main	15,54%
LEU	260	Main	LEU	291	Main	17,79%	ALA	252	Main	ASP	289	Main	15,14%
SER	180	Side	PRO	224	Main	17,39%	ASP	286	Main	VAL	323	Main	14,99%
ASN	326	Main	GLU	247	Side	16,84%	THR	155	Main	TRP	192	Main	14,94%
ILE	184	Main	ASP	182	Side	16,69%	ARG	133	Main	ASP	170	Side	14,54%
GLY	221	Main	SER	218	Main	16,64%	ALA	196	Main	LEU	233	Main	14,39%
TRP	132	Side	LEU	254	Main	16,39%	LEU	290	Main	LYS	327	Main	14,09%
LYS	135	Main	ASP	131	Main	16,39%	SER	203	Main	TRP	240	Main	13,99%
ALA	255	Main	ALA	251	Main	16,29%	ASN	331	Side	GLN	368	Main	13,99%
ALA	252	Main	ASP	248	Main	15,39%	HIP	287	Main	VAL	324	Main	13,49%
GLN	332	Side	ASN	331	Side	15,19%	GLY	221	Main	SER	258	Main	13,44%
ILE	319	Main	ILE	305	Main	15,09%	ASN	257	Side	TYR	294	Main	13,39%
ASP	185	Main	ASP	182	Side	14,64%	SER	172	Side	GLU	209	Side	12,89%
LEU	290	Main	LYS	306	Main	13,54%	ASN	202	Side	ASN	239	Main	12,84%
ASN	273	Side	TYR	272	Main	13,49%	TYR	346	Main	GLU	383	Main	12,69%
HIP	231	Side	GLN	227	Main	13,24%	SER	308	Side	GLN	345	Side	12,09%
GLU	222	Main	ASN	220	Side	13,19%	SER	268	Main	ASP	305	Side	12,09%
SER	203	Main	TRP	199	Main	13,04%	ALA	251	Main	GLU	288	Main	11,99%
VAL	201	Main	PHE	197	Main	12,99%	TYR	302	Main	ASN	339	Main	11,84%
LEU	345	Main	GLU	343	Side	12,49%	GLU	222	Main	ASN	259	Side	11,79%
ASN	202	Side	ASN	198	Main	12,39%	GLY	206	Main	ILE	243	Main	10,94%
ASN	207	Main	ASN	204	Main	11,79%	ASP	248	Main	ASP	285	Side	10,89%
ALA	137	Main	TRP	132	Main	11,59%	ASN	344	Side	GLU	381	Side	10,84%
ALA	196	Main	LEU	192	Main	11,54%	HIP	231	Side	SER	268	Main	10,69%
SER	218	Main	GLN	176	Side	11,44%	SER	180	Side	GLN	217	Main	10,69%
CYX	188	Main	GLN	223	Side	11,39%	SER	310	Side	TRP	347	Main	10,14%
TYR	302	Main	ASN	295	Main	11,34%	TRP	132	Side	LEU	169	Main	10,09%
GLN	162	Side	ASN	257	Main	11,19%	SER	310	Main	LYS	347	Main	9,95%

ASP	248	Main	ASP	246	Side	11,14%
VAL	179	Main	GLU	175	Main	10,54%
ASN	257	Side	TYR	253	Main	10,14%
TYR	214	Main	THR	210	Main	10,04%
GLN	227	Side	ASN	229	Main	9,95%
ALA	251	Main	GLU	247	Main	9,95%
SER	180	Side	GLN	176	Main	9,90%
GLY	157	Main	PHE	153	Main	9,65%
GLY	317	Main	TRP	313	Main	9,65%
TYR	346	Main	GLU	343	Main	9,30%
GLY	206	Main	ILE	200	Main	9,05%
SER	149	Side	TYR	216	Side	8,95%
GLN	164	Side	ALA	137	Main	8,45%
MET	330	Main	GLN	327	Main	8,40%
SER	154	Side	ILE	262	Main	8,35%
GLY	189	Main	PHE	186	Main	8,25%
SER	310	Main	LYS	142	Main	8,10%
ALA	261	Main	SER	335	Main	8,00%
SER	298	Main	ASN	295	Side	7,85%
ARG	133	Main	ASP	131	Side	7,45%
ASN	207	Side	ASN	204	Main	7,40%
ASN	198	Side	ASP	194	Main	7,30%
TRP	151	Side	GLY	187	Main	7,20%
HIP	231	Side	SER	213	Main	7,10%
ASP	265	Main	CYX	328	Main	6,95%
GLN	164	Main	GLU	160	Main	6,95%
ASN	295	Side	TYR	302	Side	6,20%
GLN	245	Side	GLN	332	Side	6,15%
ASN	168	Side	GLY	340	Main	5,70%
TRP	313	Side	PHE	269	Main	5,45%
TYR	318	Main	GLU	315	Main	5,35%
GLN	164	Side	ASN	168	Main	5,15%
LEU	192	Main	ASP	185	Side	5,05%
ILE	262	Main	VAL	289	Main	5,05%
ALA	166	Main	GLN	162	Main	5,05%
GLY	340	Main	ALA	236	Main	5,05%
GLU	134	Main	ASP	131	Side	5,05%

MET	330	Main	GLN	367	Main	9,90%
SER	298	Side	ASN	335	Side	9,30%
ILE	319	Main	ILE	356	Main	9,25%
GLN	162	Side	ASN	199	Main	9,10%
GLN	332	Side	ASN	369	Side	8,95%
LEU	192	Main	ASP	229	Side	8,80%
TRP	199	Side	THR	236	Side	8,60%
TYR	346	Main	PRO	383	Main	8,55%
SER	154	Side	ILE	191	Main	8,50%
ASN	207	Main	ASN	244	Main	8,30%
GLY	157	Main	PHE	194	Main	8,25%
GLN	332	Side	LEU	369	Main	7,95%
GLY	317	Main	TRP	354	Main	7,85%
GLY	189	Main	PHE	226	Main	7,70%
ASN	207	Side	ASN	244	Main	7,70%
LYS	306	Side	THR	343	Main	7,45%
TYR	214	Main	THR	251	Main	7,45%
ASN	273	Side	TYR	310	Main	7,25%
LEU	243	Main	VAL	280	Main	7,05%
LEU	178	Main	GLU	215	Main	6,95%
TYR	318	Main	GLU	355	Main	6,65%
SER	336	Main	VAL	373	Main	6,55%
ALA	333	Main	MET	370	Main	6,40%
ILE	262	Main	VAL	299	Main	6,25%
ALA	261	Main	SER	298	Main	6,15%
SER	298	Main	ASN	335	Side	6,10%
SER	218	Main	GLN	255	Side	5,90%
GLY	167	Main	GLN	204	Main	5,90%
ASN	295	Side	TYR	332	Side	5,85%
LEU	345	Main	PRO	382	Main	5,80%
SER	218	Side	VAL	255	Main	5,70%
GLN	332	Main	LEU	369	Main	5,40%
ILE	321	Main	TRP	358	Main	5,25%

Table S6a: Relative occurrence of hydrogen bonds between pro- and catalytic domain. Four respective MD simulations at pH 4 (pH4_1 and pH4_2, see Table S6b for pH4_3 and pH4_4) were carried out. `Main' refers to interactions of the backbone. 'Side' indicates interactions of side chains. Occurrences below 5% were left out for clarity.

pH4_1							pH 4_2						
donor			acceptor			occupancy	donor			acceptor			occupancy
TYR	101	Side	CYM	150	Side	64,50%	THR	83	Main	GLY	274	Main	43,50%
ASH	271	Side	GLN	71	Side	63,10%	ASH	271	Side	ASN	68	Side	41,40%
SER	268	Side	GLN	75	Side	57,50%	THR	87	Side	MET	270	Main	39,25%
SER	90	Side	MET	270	Main	46,15%	GLY	85	Main	ASH	271	Main	38,65%
VAL	119	Main	ASP	242	Main	44,55%	SER	279	Main	ASN	79	Side	36,25%
ASN	79	Side	SER	279	Main	38,85%	VAL	119	Main	ASP	242	Main	33,60%
THR	83	Main	GLY	274	Main	36,10%	SER	90	Side	ASH	271	Side	32,15%
GLY	85	Main	ASH	271	Main	34,95%	ILE	276	Main	TYR	81	Main	25,70%
SER	279	Main	ASN	79	Side	26,20%	ARG	102	Side	ASP	265	Side	20,35%
ASP	242	Main	LYS	117	Main	26,20%	TYR	107	Side	ILE	184	Main	19,85%
GLN	71	Side	THR	267	Main	22,75%	ASP	242	Main	LYS	117	Main	18,00%
LYS	117	Main	ASP	242	Side	21,15%	ASN	273	Side	VAL	86	Main	17,80%
ASN	273	Side	VAL	86	Main	19,05%	ARG	100	Side	GLN	146	Side	17,15%
GLN	75	Side	SER	268	Main	18,30%	LYS	117	Main	ASP	242	Side	15,85%
GLN	75	Side	ASH	271	Side	17,15%	ASN	273	Main	THR	83	Main	12,10%
ARG	114	Side	ASN	195	Side	16,85%	LYS	117	Side	HIP	240	Main	8,85%
TYR	107	Side	ILE	184	Main	16,55%	ARG	116	Side	ASP	242	Side	8,00%
THR	278	Main	ASN	79	Side	16,10%	THR	278	Main	ASN	79	Side	7,85%
LYS	117	Side	ASP	239	Side	13,30%	LYS	117	Side	ASP	239	Side	6,75%
GLN	245	Side	GLN	112	Side	12,80%	TYR	107	Side	GLY	189	Main	6,50%
THR	281	Side	GLN	75	Side	12,60%	ASN	79	Side	SER	279	Main	6,35%
ASN	120	Main	TYR	346	Main	12,25%	ARG	116	Side	VAL	334	Main	5,95%
ASN	273	Main	THR	83	Main	12,20%	GLN	245	Side	GLN	112	Side	5,30%
THR	281	Main	GLN	75	Side	11,90%	GLN	112	Side	GLN	332	Side	5,15%
ILE	276	Main	TYR	81	Main	11,65%							
GLN	284	Side	ALA	105	Main	9,15%							
ARG	116	Side	VAL	334	Main	7,25%							
THR	281	Side	GLN	71	Side	6,85%							
GLN	112	Side	GLN	245	Side	6,35%							
ARG	98	Side	GLH	283	Side	5,85%							
GLY	104	Main	GLY	189	Main	5,70%							
GLY	124	Main	ALA	252	Main	5,00%							

Table S6b: Relative occurrence of hydrogen bonds between pro- and catalytic domain. Four respective MD simulations at pH 4 (pH4_3 and pH4_4, see Table S6a for pH4_1 and pH4_2) were carried out. `Main' refers to interactions of the backbone. 'Side' indicates interactions of side chains. Occurrences below 5% were left out for clarity.

pH 4_3							pH 4_4						
donor			acceptor			occupancy	donor			acceptor			occupancy
THR	87	Side	MET	270	Main	57,47%	SER	279	Main	ASN	79	Side	38,43%
THR	83	Main	GLY	274	Main	41,98%	GLY	85	Main	ASH	271	Main	36,78%
GLY	85	Main	ASH	271	Main	38,63%	THR	83	Main	GLY	274	Main	35,83%
SER	279	Main	ASN	79	Side	34,98%	VAL	119	Main	ASP	242	Main	32,53%
VAL	119	Main	ASP	242	Main	32,23%	ASN	273	Side	VAL	86	Main	28,44%
ASN	79	Side	SER	279	Main	23,34%	ILE	276	Main	TYR	81	Main	17,24%
ASH	271	Side	ASN	68	Side	21,94%	ASN	273	Main	THR	83	Main	16,94%
THR	122	Side	GLN	348	Main	21,74%	THR	87	Side	MET	270	Main	15,39%
ASN	273	Side	VAL	86	Main	20,94%	ASN	79	Side	SER	279	Main	12,94%
TYR	107	Side	ILE	184	Main	20,94%	SER	268	Side	GLN	75	Side	12,04%
ASN	273	Main	THR	83	Main	14,39%	GLN	112	Side	GLN	332	Side	10,54%
SER	268	Side	GLN	75	Side	13,94%	THR	278	Main	ASN	79	Side	8,65%
THR	278	Main	ASN	79	Side	13,84%	GLN	75	Side	THR	281	Side	8,65%
ILE	276	Main	TYR	81	Main	13,49%	TYR	107	Side	ILE	184	Main	7,80%
SER	90	Side	ASH	271	Side	13,29%	TYR	107	Side	GLY	189	Main	6,05%
ASN	103	Side	GLY	189	Main	11,74%	ARG	116	Side	VAL	334	Main	5,55%
GLN	112	Side	GLN	332	Side	9,60%	ARG	100	Side	GLN	146	Side	5,50%
THR	122	Side	GLN	348	Side	9,60%	TYR	101	Side	ALA	266	Main	5,00%
ARG	100	Side	GLN	146	Side	9,30%							
ARG	116	Side	ASP	242	Side	9,00%							
THR	122	Main	GLN	348	Side	6,50%							
TYR	48	Side	MET	312	Main	6,15%							
GLN	75	Side	THR	281	Side	6,15%							
ASH	271	Side	GLN	71	Side	5,40%							

Table S7a: Relative occurrence of hydrogen bonds between pro- and catalytic domain. Four respective MD simulations at pH 8 (pH8_1 and pH8_2, see Table 7b for pH8_3 and pH8_4) were carried out. `Main' refers to interactions of the backbone. 'Side' indicates interactions of side chains. Occurrences below 5% were left out for clarity.

pH_8_1							pH 8_2						
donor			acceptor			occupancy	donor			acceptor			occupancy
GLN	71	Side	ASP	271	Side	66,50%	GLN	75	Side	ASP	271	Side	60,30%
THR	87	Side	MET	270	Main	43,95%	ARG	116	Side	ASP	242	Side	52,35%
THR	83	Main	GLY	274	Main	39,15%	THR	83	Main	GLY	274	Main	41,35%
SER	279	Main	ASN	79	Side	36,40%	VAL	119	Main	ASP	242	Main	41,10%
VAL	119	Main	ASP	242	Main	34,05%	ASP	242	Main	LYS	117	Main	37,95%
GLY	85	Main	ASP	271	Main	33,05%	THR	87	Side	MET	270	Main	37,65%
ARG	116	Side	VAL	334	Main	29,15%	LYS	117	Main	ASP	242	Side	34,70%
ASN	273	Side	VAL	86	Main	27,30%	SER	279	Main	ASN	79	Side	27,05%

ASN	68	Side	ASP	271	Side	25,45%	TYR	107	Side	ASP	185	Side	25,60%
GLN	75	Side	ASP	271	Side	24,20%	ILE	276	Main	TYR	81	Main	24,30%
ILE	276	Main	TYR	81	Main	23,70%	TYR	48	Side	MET	312	Main	23,90%
ARG	114	Side	ASP	242	Side	21,85%	GLY	85	Main	ASP	271	Main	23,80%
TYR	107	Side	ASP	185	Side	21,05%	ARG	116	Side	ASP	194	Side	22,05%
ARG	116	Side	ASP	194	Side	19,60%	ARG	98	Side	THR	267	Side	20,35%
THR	281	Side	GLN	75	Side	18,35%	ASN	273	Side	VAL	86	Main	17,85%
ASN	273	Main	THR	83	Main	11,80%	ARG	114	Side	ASN	195	Side	17,55%
ASN	103	Side	GLY	189	Main	11,20%	ASN	103	Side	GLY	189	Main	16,45%
ARG	98	Side	THR	267	Side	9,75%	ARG	114	Side	ASP	194	Side	15,05%
ARG	114	Side	ASP	194	Side	9,05%	LYS	117	Side	HIE	240	Main	14,35%
LYS	117	Main	ASP	242	Side	7,25%	THR	281	Side	GLN	75	Side	14,30%
GLN	112	Side	GLN	332	Main	6,70%	ARG	98	Side	ASP	286	Side	13,00%
ASN	79	Side	SER	279	Main	5,05%	ASN	273	Main	THR	83	Main	9,20%
GLN	112	Side	GLN	332	Side	5,00%	GLN	71	Side	ASP	271	Side	8,90%
							LYS	117	Side	ASP	239	Side	8,65%
							GLN	75	Side	SER	268	Main	7,60%
							ASN	79	Side	SER	279	Main	7,50%
							ARG	100	Side	GLN	146	Side	7,15%
							ARG	125	Side	GLU	256	Side	6,55%
							SER	268	Side	GLN	75	Side	5,70%
							GLN	75	Side	SER	268	Side	5,40%
							GLN	112	Side	GLN	332	Side	5,00%

Table S7b: Relative occurrence of hydrogen bonds between pro- and catalytic domain. Four respective MD simulations at pH 8 (pH8_3 and pH8_4, see Table S7a for pH8_1 and pH8_2) were carried out. `Main' refers to interactions of the backbone. 'Side' indicates interactions of side chains. Occurrences below 5% were left out for clarity.

pH 8_3							pH 8_4						
donor			acceptor			occupancy	donor			acceptor			occupancy
ASN	68	Side	ASP	271	Side	70,51%	THR	87	Side	MET	270	Main	64,42%
GLN	75	Side	ASP	271	Side	51,67%	ARG	116	Side	ASP	194	Side	50,07%
ARG	116	Side	ASP	194	Side	49,58%	THR	83	Main	GLY	274	Main	41,83%
THR	87	Side	MET	270	Main	48,03%	ASN	68	Side	ASP	271	Side	39,38%
VAL	119	Main	ASP	242	Main	42,78%	ARG	116	Side	VAL	334	Main	36,68%
ARG	125	Side	GLU	256	Side	37,68%	GLN	75	Side	ASP	271	Side	27,89%
THR	83	Main	GLY	274	Main	37,58%	THR	83	Side	ASN	273	Side	27,69%
SER	279	Main	ASN	79	Side	33,23%	ASN	273	Main	THR	83	Main	25,74%
ARG	94	Side	ASP	271	Side	30,38%	ILE	276	Main	TYR	81	Main	25,14%
TYR	107	Side	ASP	185	Side	26,34%	ARG	94	Side	ASP	271	Side	24,94%
LYS	117	Main	ASP	242	Side	24,69%	GLY	85	Main	ASP	271	Main	24,84%
ASN	273	Side	VAL	86	Main	19,49%	THR	122	Side	GLU	256	Side	23,99%
GLY	85	Main	ASP	271	Main	17,59%	LYS	117	Main	ASP	242	Side	19,94%
GLN	71	Side	ASP	271	Side	16,49%	GLN	71	Side	ASP	271	Side	19,54%
ILE	276	Main	TYR	81	Main	16,19%	LYS	117	Side	ASP	242	Side	17,04%
ASN	103	Side	GLY	189	Main	15,29%	SER	279	Main	ASN	79	Side	14,04%

ASP	242	Main	LYS	117	Main	10,84%	ARG	102	Side	GLU	283	Side	13,04%
ARG	102	Side	ASP	286	Side	9,65%	GLN	75	Side	SER	268	Main	11,49%
ARG	116	Side	VAL	334	Main	9,45%	THR	122	Main	ALA	252	Main	11,44%
ARG	114	Side	ASN	195	Side	8,35%	ARG	114	Side	ASN	195	Side	10,59%
ASN	273	Main	THR	83	Main	7,70%	TYR	107	Side	GLY	189	Main	8,75%
ASN	79	Side	SER	279	Main	7,10%	GLN	112	Side	GLN	332	Main	7,75%
ARG	102	Side	GLN	284	Side	6,60%	ASN	273	Side	VAL	86	Main	7,60%
GLN	75	Side	SER	268	Main	6,45%	ARG	114	Side	ASP	242	Side	7,60%
							TYR	48	Side	MET	312	Main	7,10%
							ARG	125	Side	GLU	256	Side	6,25%
							ASN	103	Side	GLY	189	Main	5,35%
							LYS	117	Main	ASP	242	Main	5,30%

References

1. Caffrey, C. R. *et al.* Active site mapping, biochemical properties and subcellular localization of rhodesain, the major cysteine protease of Trypanosoma brucei rhodesiense. *Mol. Biochem. Parasitol.* **118**, 61–73 (2001).
2. Kerr, I. D. *et al.* Vinyl sulfones as antiparasitic agents and a structural basis for drug design. *J. Biol. Chem.* **284**, 25697–25703 (2009).
3. Micsonai, A. *et al.* Accurate secondary structure prediction and fold recognition for circular dichroism spectroscopy. 1–9 (2015).
